# Structure, Function and Regulation of a Second Pyruvate Kinase Isozyme in *Pseudomonas aeruginosa*

**DOI:** 10.3389/fmicb.2021.790742

**Published:** 2021-11-16

**Authors:** Yassmin Abdelhamid, Meng Wang, Susannah L. Parkhill, Paul Brear, Xavier Chee, Taufiq Rahman, Martin Welch

**Affiliations:** ^1^Department of Biochemistry, University of Cambridge, Cambridge, United Kingdom; ^2^Department of Pharmacology, University of Cambridge, Cambridge, United Kingdom

**Keywords:** bacterial metabolism, Entner-Doudoroff pathway, glycolysis, *Pseudomonas aeruginosa*, pyruvate kinase, pykF, x-ray crystallography

## Abstract

*Pseudomonas aeruginosa* (PA) depends on the Entner-Doudoroff pathway (EDP) for glycolysis. The main enzymatic regulator in the lower half of the EDP is pyruvate kinase. PA contains genes that encode two isoforms of pyruvate kinase, denoted PykA_PA_ and PykF_PA_. In other well-characterized organisms containing two pyruvate kinase isoforms (such as *Escherichia coli*) each isozyme is differentially regulated. The structure, function and regulation of PykA_PA_ has been previously characterized in detail, so in this work, we set out to assess the biochemical and structural properties of the PykF_PA_ isozyme. We show that *pykF*_PA_ expression is induced in the presence of the diureide, allantoin. In spite of their relatively low amino acid sequence identity, PykA_PA_ and PykF_PA_ display broadly comparable kinetic parameters, and are allosterically regulated by a very similar set of metabolites. However, the x-ray crystal structure of PykF_PA_ revealed significant differences compared with PykA_PA_. Notably, although the main allosteric regulator binding-site of PykF_PA_ was empty, the “ring loop” covering the site adopted a partially closed conformation. Site-directed mutation of the proline residues flanking the ring loop yielded apparent “locked on” and “locked off” allosteric activation phenotypes, depending on the residue mutated. Analysis of PykF_PA_ inter-protomer interactions supports a model in which the conformational transition(s) accompanying allosteric activation involve re-orientation of the A and B domains of the enzyme and subsequent closure of the active site.

## Introduction

*Pseudomonas aeruginosa* (PA) is a well-known opportunistic human pathogen and is associated with airway, burn wound, opthalmic and other soft-tissue infections (Preston et al., 1997; Martínez-Solano et al., 2008; Turner et al., 2014). Although it can readily consume glucose, PA does not encode the Embden-Meyerhof-Parnas (EMP) pathway enzyme, phosphofructokinase, and is therefore entirely reliant upon the Entner-Doudoroff pathway (EDP) for glycolysis (Kersters and De Ley, 1968; Lessie and Phibbs, 1984; Temple et al., 1998). The enzymatic logic of the EMP and the EDP are broadly similar; glucose is taken up and phosphorylated, and, following a series of downstream transformations distinct to each pathway, the product is cleaved in a reverse aldol condensation reaction to yield two three-carbon compounds. However, the substrate of the aldol cleavage is different; in the case of the EMP, fructose 1,6-*bis*phosphate is cleaved to yield glyceraldehyde 3-phosphate (G3P) and dihydroxyacetone phosphate, whereas in the EDP, 2-*keto*-3-deoxy-6-phosphogluconate (KDPG) is cleaved to yield G3P and pyruvate (Kovachevich and Wood, 1955a,b; Drechsler et al., 1959; Peekhaus and Conway, 1998). In both the EMP and the EDP, the metabolic fate of G3P following the aldol cleavage step is identical. The pathways can therefore be conveniently divided into distinct “upper reactions” (which precede the aldol cleavage) and a common set of “lower reactions.”

Pyruvate kinase [ATP:pyruvate 2-*O*-phosphotransferase (EC 2.7.1.40)] catalyzes the interconversion of phospho*enol*pyruvate and pyruvate in the final reaction of the “lower half” of EDP and EMP glycolysis, and is widely regarded as the main regulatory enzyme for this sequence of reactions (Rose, 1970; Kayne, 1973; Seeholzer et al., 1991; Al-Zaid Siddiquee et al., 2004; Bücker et al., 2014; Noy et al., 2016).


phosphoenolpyruvate+ADP↔pyruvate+ATP

*Pseudomonas aeruginosa* is among a subset of bacteria that express two distinct pyruvate kinase isoforms, denoted PykA and PykF (Waygood et al., 1975, 1976; Garcia-Olalla and Garrido-Pertierra, 1987; Hofmann et al., 2013; Abdelhamid et al., 2019). Both isozymes catalyze the same reaction. We previously demonstrated that PykA displays potent K-type allosteric activation by glucose 6-phosphate (G6P), fructose 6-phosphate (F6P), G3P and by intermediates of the reductive pentose phosphate pathway (PPP) (Abdelhamid et al., 2019). It is important to note here that in PA, the upper half of the EDP and the upper half of the gluconeogenic pathway (encoding the aldolase, fructose 1,6-*bis*phosphatase, and phosphoglucoisomerase) do not operate as essentially parallel “contraflow” reactions, but instead, engage in an integrated cyclical series of reactions; the “Entner-Doudoroff-Embden-Meyerhof-Parnas” (EDEMP) cycle (Nikel et al., 2015; Kohlstedt and Wittmann, 2019). The PPP also feeds intermediates into the EDEMP cycle. Given that PPP intermediates strongly activate PykA, this suggests that flux through the lower half of the EDP is coordinated with the level of intermediates in the EDEMP cycle. Interestingly, the G6P-binding site in *P. aeruginosa* PykA (hereafter, PykA_PA_) is clearly distinct from the G6P-binding site in pyruvate kinase from *Mycobacterium tuberculosis*, indicating remarkable plasticity in the mechanism(s) underpinning allosteric regulation in each enzyme (Zhong et al., 2017; Abdelhamid et al., 2019). On the other hand, the structure, activity and regulation of *P. aeruginosa* PykF (hereafter, PykF_PA_) has not yet been characterized.

Like PA, *Escherichia coli* also contains genes that encode PykA and PykF isozymes. In *E. coli*, both isozymes are expressed, although PykF is generally considered to be the dominant isozyme during aerobic growth, whereas PykA appears to play an important role following oxygen limitation (Ponce et al., 1995; Zhao et al., 2017). The *E. coli* enzymes are differentially regulated; fructose 1,6-*bis*phosphate strongly activates PykF, whereas ribose 5-phosphate (R5P) and adenosine 5′-monophosphate (AMP) activate PykA. However, this functional distinction does not easily translate to “sequence space” and there are no obvious sequence motifs that can be used to differentiate these two classes of isozyme. This raises the question of whether PykF_PA_ might be regulated differently compared with PykA_PA_.

PykF_PA_ shares just 37% amino acid identity with PykA_PA_, and 36% identity with *E. coli* PykF. Given the relatively low level of similarity between PykF_PA_ and other well-characterized pyruvate kinases, and the lack of functional insight that can be gleaned from sequence comparisons alone, we set out here to investigate the biochemical and structural properties of this second pyruvate kinase isozyme in PA. Surprisingly, and although PykF_PA_ was regulated by a broadly similar set of compounds as PykA_PA_, its structure–especially around the likely allosteric site for G6P–was different. Structure-guided site-directed mutagenesis of some of the key residues around the sugar ring loop which “guards” the G6P-binding site revealed unexpected subtlety in the allosteric mechanism of the enzyme. Finally, we show that the inter-protomer interfaces in PykF_PA_ are very different from those in PK enzymes from other structurally characterized species, indicative of a potentially novel mechanism underpinning cooperative transitions.

## Results

### Expression of PykF_PA_

**FIGURE 1:**
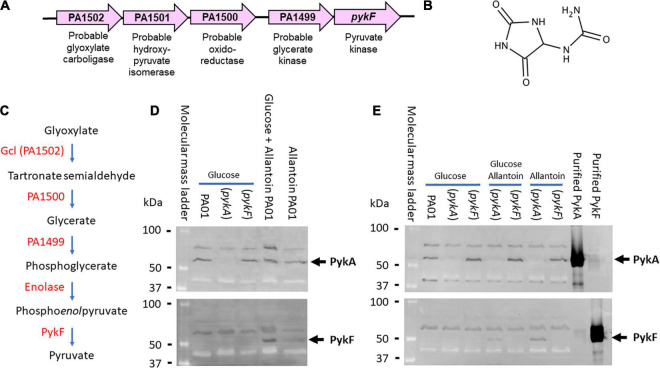
Genetic context and expression of PykF. **(A)** ORFs associated with the PA1489-PA1502 cluster and their predicted function. **(B)** Structure of allantoin. **(C)** Predicted metabolic reactions catalyzed by each encoded enzyme in the gene cluster. Note that glyoxylate and urea are the breakdown products of allantoin degradation. **(D)** PykF is not expressed in wild-type *P. aeruginosa* in the presence of glucose but is expressed in the presence of allantoin. The figure shows western blots of cell extracts of the indicated *P. aeruginosa* derivatives (wild-type PAO1, *pykA* mutant, or *pykF* mutant, as indicated) obtained after overnight growth in M9 medium containing glucose, glucose plus allantoin, or allantoin alone. The blot in the upper panel was probed with anti-PykA antibodies and the blot in the lower panel was probed with anti-PykF antibodies. The identity of the cross-reacting bands with higher molecular mass than PykA or PykF in each panel is not known, but their presence in the *pykA* and *pykF* mutant extracts indicates that they are unrelated to these pyruvate kinases. **(E)** Allantoin-induced PykF expression is abolished in a *pykF* mutant. The figure shows a western blot of cell extracts from a *pykA* or a *pykF* mutant (as indicated) grown in the presence or absence of glucose and/or allantoin (as indicated). To confirm minimal cross-reactivity of the antibodies, purified PykA or PykF (as indicated) were loaded on the right-hand side of each blot.

*PykF* (PA1498) is the terminal ORF in an uncharacterized cluster of five ORFs (PA1498-PA1502, Figure 1A). We previously demonstrated that there is no appreciable PykF expression during growth on glucose, acetate or glycerol as sole carbon sources (Abdelhamid et al., 2019). However, the presence of a probable glyoxylate carboligase (PA1502) and a putative tartronate semialdehyde reductase (PA1500) in the same gene cluster as *pykF* led us to suspect that the cluster may be involved in the terminal steps of allantoin (glyoxyldiureide, Figures 1B,C) catabolism (Cusa et al., 1999). We therefore wondered whether PykF expression might be induced in the presence of allantoin. To test this, we grew cultures (separately) of wild-type PA (strain PAO1), an isogenic *pykA* mutant, and an isogenic *pykF* mutant, in M9 minimal medium containing either glucose, allantoin, or glucose plus allantoin as a sole carbon source. Aliquots of the cultures were analyzed by Western blotting using antibodies raised against purified PykA or purified PykF, as previously described (Abdelhamid et al., 2019). PykA was expressed during growth on all of the tested carbon sources in the wild-type and in the *pykF* mutant, but was not detectable in the *pykA* mutant, as expected (Figure 1D). PykF was undetectable in cells grown on glucose as a sole carbon source [as previously reported (Abdelhamid et al., 2019)] but was expressed in cells grown on media containing allantoin (Figure 1D). This expression of PykF in the presence of allantoin was abolished in the *pykF* mutant, as expected (Figure 1E). We conclude that *pykF* expression appears to be induced in the presence of allantoin (although we note that this does not exclude the possibility that this cluster of genes may also play a role(s) in other aspects of *P. aeruginosa* physiology too). Interestingly, the *pykF* mutant displayed a marked growth defect when grown on allantoin as a sole carbon source (Supplementary Figure 1) whereas growth of the *pykA* mutant was unaffected. This suggests that PykA (which is abundantly expressed during growth on allantoin; Figures 1D,E) cannot fully substitute for PykF under these circumstances, although we cannot rule out the possibility that the Tn insertion in this terminal ORF may have polar effects on the 3′ end of the cluster too. Current efforts are aimed at confirming the function of each of the other enzymes in the PA1498-PA1502 cluster, and in identifying how allantoin might regulate this cluster at a genetic level. However, the conditional expression of PykF raises the question of why this additional pyruvate kinase isoform is needed at all, especially given the apparently constitutive expression of PykA. One possibility is that PykF_PA_ displays different kinetic or regulatory properties compared with PykA_PA_, so this is what we investigated next.

### Kinetic Properties of PykF_PA_

**FIGURE 2:**
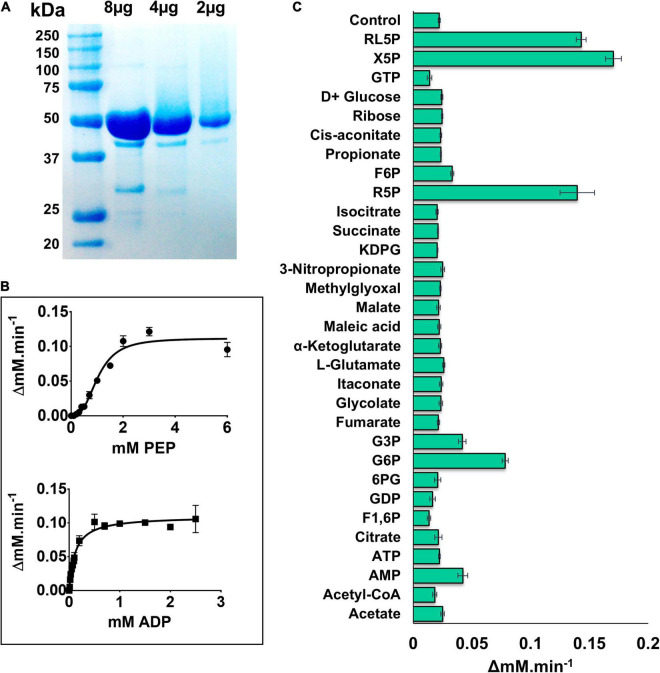
Kinetic characterization of PykF. **(A)** SDS-PAGE gel showing purified, untagged PykF (51.5 kDa). The gel was prepared with 12% (w/v) acrylamide and stained with Coomassie Brilliant Blue G250 [The minor bands at around 30 and 40 kDa molecular mass correspond to degradation products of PykF]. **(B)** PykF kinetics with respect to PEP and ADP. The PEP titration was performed using 2 mM ADP (i.e., a saturating concentration) whereas the ADP titration was performed using 5 mM PEP (also a saturating concentration). The *R*^2^ values for the curve fit to the PEP and ADP titrations were 0.96 in both cases. The 95% confidence interval estimates for the kinetic parameters are shown in Supplementary Table 4. **(C)** The effect of different metabolic regulators on PykF activity at low [PEP] (0.3 mM) and 2 mM ADP. Putative regulators were added at 1 mM final concentration, except for R5P, X5P, and RL5P which were used at 0.15, 0.5, and 0.5, respectively. The aim of this experiment was to identify potential activators. Data in panels **(B,C)** represent the mean and standard deviation of three independent experiments.

PykF_PA_ was purified as previously described (Abdelhamid et al., 2019). Analytical ultracentrifugation (AUC) analysis revealed that the purified PykF_PA_ [monomeric molecular mass 51.5 kDa (Figure 2A)] is a tetramer in solution with a molecular mass of approximately 200 kDa (Supplementary Figure 2). Kinetic analyses revealed that PykF_PA_ exhibited a similar kinetic profile toward phospho*enol*pyruvate (PEP) and ADP as most other well-characterized PK enzymes (Figure 2). PykF_PA_ displayed sigmoidal kinetics in response to PEP titration, with an S_0_._5_ value of 1.03 mM and a Hill coefficient (h) of 2.82, indicative of positive homotropic cooperativity (Figure 2 and Table 1). By contrast, PykF_PA_ displayed Michaelis-Menten (hyperbolic) kinetics in response to ADP titration, with a K_*M*_ of 0.11 mM ± 0.01 (Figure 2). These S_0_._5__(PEP)_ and K_*M*__(ADP)_ values for PykF_PA_ were somewhat higher than the previously reported values for PykA_PA_ [S_0_._5__(PEP)_ = 0.67 mM and K_M__(ADP)_ = 0.07 mM (Abdelhamid et al., 2019)], suggesting that PykF_PA_ is intrinsically only slightly less active than PykA_PA_ [For further comparison, the k_cat(PEP)_ and k_cat(PEP)_/S_0_._5__(PEP)_ values for PykF_PA_ were 379 s^–1^ and 367 s^–1^ mM^–1^, whereas the corresponding values for PykA_PA_ were 432 s^–1^ and 644 s^–1^ mM^–1^, respectively].

**TABLE 1:** The effect of metabolic intermediates on PykF activity.

**PEP titration**	**No additive**	**1** **mM G6P**	**0.15** **mM R5P**	**1** **mM AMP**	**1** **mM G3P**	**0.5** **mM X5P**	**0.5** **mM RL5P**	**Pro455→ Ala No G6P**	**Pro455→ Ala** **+** **G6P**	**Pro459→ Ala No G6P**	**Pro459→ Ala** **+** **G6P**	**Pro455, Pro459 →Ala No G6P**	**Pro455, Pro459 →Ala** **+** **G6P**
S_0_._5_ (mM)	1.03 ± 0.01	0.34 ± 0.04	0.23 ± 0.03	0.56 ± 0.02	0.68 ± 0.02	0.11 ± 0.01	0.17 ± 0.01	0.48 ± 0.02	0.47 ± 0.03	1.11 ± 0.05	0.92 ± 0.07	2.05 ± 0.53	1.90 ± 0.51
Hill Coefficient (h)	2.82 ± 0.27	1.25 ± 0.18	1.47 ± 0.32	2.03 ± 0.15	2.69 ± 0.17	1.58 ± 0.21	2.15 ± 0.24	1.65 ± 0.11	1.38 ± 0.13	3.91 ± 0.66	2.63 ± 0.44	1.07 ± 0.18	1.09 ± 0.14
V_*max*_ (ΔmM.min^–1^)	0.11 ± 0.00	0.13 ± 0.01	0.14 ± 0.01	0.11 ± 0.00	0.09 ± 0.00	0.13 ± 0.00	0.13 ± 0.00	0.06 ± 0.00	0.06 ± 0.00	0.04 ± 0.00	0.04 ± 0.00	0.04 ± 0.00	0.04 ± 0.00
k_cat_ (s^–1^)	378.6	446.7	515.4	365.6	316.2	432.9	439.8	249.6	249.6	126.9	151.6	146.7	154.7
k_cat_/S_0_._5_ (s^–1^.mM^–1^)	367	1314	2241	653	465	3935	2587	522	530	114	165	72	81

*Kinetic parameters were calculated using GraphPad Prism from best-fit non-linear regression analysis of the data. Abbreviations: R5P, ribose 5-phosphate; G6P, glucose 6-phosphate; AMP, adenosine 5′ monophosphate; G3P, glyceraldehyde 3-phosphate; X5P, xylulose 5-phosphate; RL5P, ribulose 5-phosphate. k_*cat*_ was calculated using [E_*t*_] = [PykF monomer]. All regulators were added at 1 mM final concentration except R5P, X5P and RL5P which were used at 0.15, 0.5, and 0.5 mM, respectively.*

All PKs require Mg^2+^ for catalysis, and many also require K^+^ in order to achieve maximal activity (Kachmar and Boyer, 1953; Baek and Nowak, 1982). Consistent with this, purified PykF_PA_ was strongly dependent on the presence of Mg^2+^ in the assay mixtures for activity (Supplementary Figure 3A). However, monovalent cations (K^+^, NH_4_^+^, and Na^+^) did not synergize this activity, and indeed, when present at 100 mM concentration, even had a detrimental effect on the activity of PykF_PA_ (Supplementary Figures 3B,C). A similar K^+^-independent activity profile was previously observed with purified PykA_PA_ (Abdelhamid et al., 2019) and can be attributed to the presence of a lysine residue at position 74 in the sequence G_72_PKLR_76_ (PykF_PA_ numbering, Supplementary Figure 4). K^+^-dependent PK enzymes generally contain a glutamate residue at the equivalent position (Laughlin and Reed, 1997; Oria-Hernández et al., 2006). PykF_PA_ activity was also supported by Co^2+^ and, to a lesser extent, also by Mn^2+^ (*data not shown*).

### Regulation of PykF_PA_

Enteric species such as *E. coli* and *Salmonella enterica* serovar Typhimurium depend primarily on the EMP for glycolysis (Romano and Conway, 1996; Bumann and Schothorst, 2017), and also contain genes encoding two PK isoforms (Valentini et al., 1979; Garcia-Olalla and Garrido-Pertierra, 1987). The PykF isoforms from these species have been found to be strongly activated by fructose 1,6-*bis*phosphate (F1,6BP), the product of phosphofructokinase (Pfk) action (Waygood and Sanwal, 1974; Waygood et al., 1976; Garcia-Olalla and Garrido-Pertierra, 1987). However, PA exclusively uses the EDP for glycolysis, and does not encode *pfk* (although F1,6BP can be generated in this organism through gluconeogenesis). Therefore, it is plausible that PykF_PA_ is regulated differently compared with the enteric isozymes. The activity of PykF_PA_ was measured in the presence of metabolites from the EDP, EMP/gluconeogenesis pathway, TCA cycle, and PPP to identify potential regulatory molecules. PykF_PA_ was not activated by F1,6BP (Figure 2C), but was strongly activated by metabolites from the non-oxidative PPP [(xylulose 5-phosphate (X5P), ribulose 5-phosphate (RL5P), and R5P], and also, to a lesser extent, by the EDP intermediates G6P and G3P (Figure 2C). The same set of regulators also activate PykA_PA_ (Abdelhamid et al., 2019). However, there were some differences between the enzymes. For example, PykF_PA_ (but not PykA_PA_) was activated by AMP whereas F6P and KDPG, which are activators of PykA_PA_, had little effect on PykF_PA_.

**FIGURE 3:**
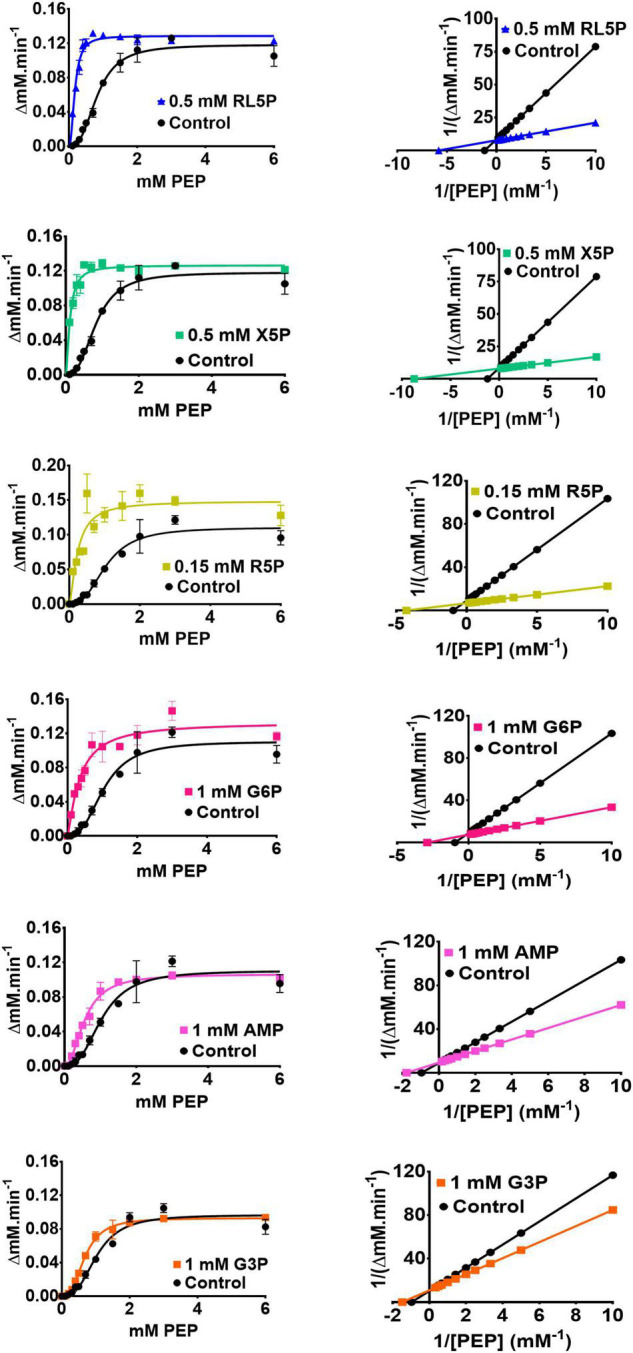
The metabolic regulation of PykF. (Left panel) Michaelis-Menten plots showing that the indicated regulators primarily convert PykF from sigmoidal to hyperbolic kinetics. Data represent the mean and standard deviation of three independent experiments. (Right panel) Lineweaver-Burk plots showing that the indicated regulators primarily act to decrease S_0_._5_ of PykF compared with the control. “Control” indicates the reaction kinetics in the absence of added regulators. The changes in PykF kinetic constants elicited by each regulator are shown in Table 1. The 95% confidence interval estimates for the kinetic parameters are provided in Supplementary Table 4.

Detailed analysis of these regulators (which all appeared to be “K-type activators,” affecting S_0_._5_ rather than k_cat_) revealed that the PPP metabolites were the most potent activators (Table 1 and Figure 3). In the presence of these compounds, the sigmoidal PEP kinetics of PykF_PA_ became more Michaelis-Menten-like (hyperbolic), as indicated by the decreased Hill coefficient (h) compared with the control. The ADP-dependency of PykF_PA_ was unaffected by these compounds (Supplementary Figure 5).

### Structural Features of PykF_PA_

**FIGURE 4:**
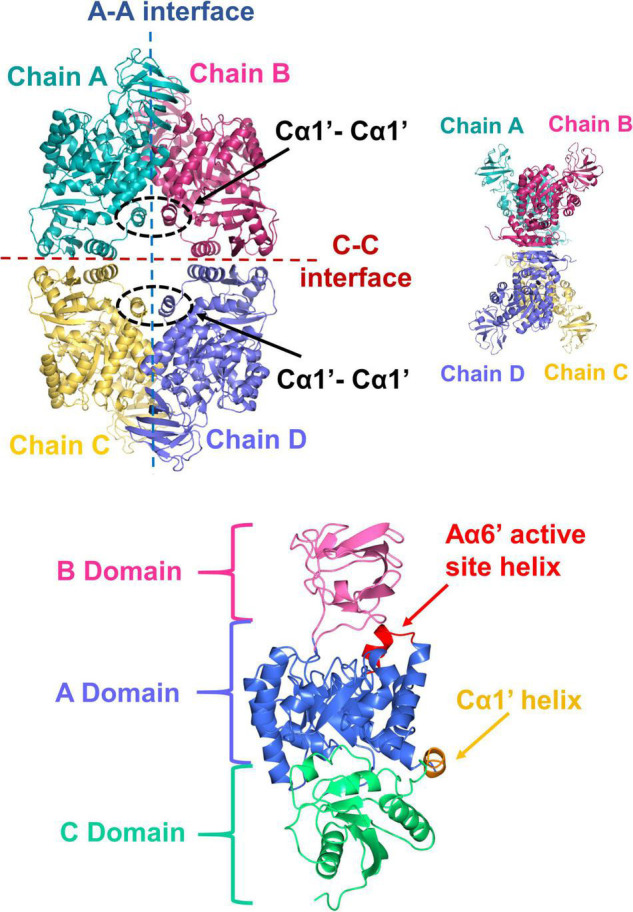
X-ray crystal structure of PykF. (Top diagrams) Front and side views of the PykF homotetramer. Chain A and B were already present in the asymmetric unit of PykF, whereas chains C and D were generated using symmetry coordinates. The A-A and the C-C interfaces are shown. The Cα1′ helices across the A-A interface are shown in dashed ovals. (Bottom diagram) Domain organization of a PykF subunit. A-, B- and C-domains of PykF are colored in blue, pink, and green, respectively. The active site helix and Cα1′ are colored in red and orange, respectively.

To further investigate the possible differences between PykA_PA_ and PykF_PA_, we determined the x-ray crystal structure of untagged full-length (477 residues) PykF_PA_ to 3.01 Å resolution (PDB 7OO1). Attempts to improve this resolution through fine screening around the best crystallization conditions, or through the use of crystallization additives, were unsuccessful. However, at 3.01 Å resolution, most of the important structural features could be assigned. The crystallization and diffraction statistics are provided in Table 2. Table 3 summarizes the main structural differences between PykF_PA_ and other published bacterial PK structures. Interestingly, PykF_PA_ has least amino acid sequence identity with pyruvate kinase isoform F from *E. coli* (PykF_EC_) and is structurally different to PykA_PA_. The asymmetric unit of PykF_PA_ consisted of two protomers (chain A and chain B) and a complete tetramer was generated by symmetry with chains C and D (Figure 4). Each protomer comprised three domains (denoted A, B, and C) and the tetramer contained four inter-protomer interfaces; two A-A interfaces (between adjacent A-domains), and two C-C interfaces (between adjacent C-domains) (Figure 4). The enzyme was modeled in the apo-form and attempts at obtaining diffracting crystals with bound regulator molecules were unsuccessful.

**TABLE 2:** Crystallographic data collection and refinement statistics of PykF.

**PDB code**	**7OO1**
**Synchrotron/X-ray source**	Diamond Light Source
Beamline	IO4-1
**Data collection**	
Wavelength (Å)	0.9159
Resolution range (Å)	115.14–3.01 (3.09–3.01)
Space group	*P*3 2 1
Unit cell	
*a*, *b*, *c*, (Å)	169.05, 169.05, 115.11
α, β, γ (°)	90, 90, 120
Total reflections	1772874
Unique reflections	37859
Multiplicity	46.8 (21.2)
Completeness (%)	99.7 (94.0)
Mean *I*/sigma (*I*)	16.3 (1.2)
Wilson B-factor	115.96
R-merge	0.139 (3.01)
R-meas	0.140 (3.09)
CC1/2	1.000 (0.85)
**Refinement**	
Resolution range (High res) (Å)	73.20–3.01 (3.09–3.01)
Reflections used in refinement	37564 (2728)
Reflections used for R-free	1859 (108)
R-work	0.24 (0.51)
R-free	0.29 (0.47)
Number of molecules in the ASU:	2
Number of non-hydrogen atoms	
Macromolecules	7213
Ligands	N/A
Protein residues	
RMS (bonds) (Å)	0.01
RMS (angles) (°)	1.6
Ramachandran favored (%)	94
Ramachandran allowed (%)	6.0
Ramachandran outliers (%)	0.1
Average B-factor	
Macromolecules	124
Ligands	N/A
Solvent	N/A

*Values in parentheses are for the highest resolution shell. N/A: Not applicable.*

**TABLE 3:** A comparison between PykF_PA_ and other bacterial pyruvate kinase structures in the Protein Data Bank.

	**Amino acid sequence identity with PykF_PA_**	**Cα1**′ **helix (or similar)**	**Elongated Aα5-Aβ5 loop at the active site**	**Extra C-terminal sequence**
*P. aeruginosa* PykF (Q9I3L4)	100%	+	−	−
*B. stearothermophilus* Pyk (Q02499)	42%	+	−	+
*M. tuberculosis* Pyk (P9WKE5)	40.5%	−	−	−
*S. aureus* Pyk (Q6GG09)	37.6%	+	−	+
*P. aeruginosa* PykA (Q9HW72)	37%	−	+	−
*E. coli* PykF (P0AD61)	36%	−	−	−

*The pyruvate kinase isoforms are identified using their gene names and UniProt IDs.*

The A-domain comprises an eight α/β TIM barrel-like structure with the α-helices spanning around a core of β-strands. Similar to PykA_PA_, Aα6 and Aα8 are preceded by shorter helical segments; denoted as Aα6′ and Aα8′, respectively. The Aα6′ helix contains the active site signature motif (M_238_VARGDLGVE_247_) (Supplementary Figure 4). As in other pyruvate kinases, the A domain was flanked on the C-terminal side by the B domain and on the N-terminal side by the C-domain. The B-domain comprises seven β-strands and a small α-helix, whereas the C-domain is formed of four α-helices alternating with five β-strands. Although the arrangement of secondary structures in the C-domain of PykF_PA_ is generally similar to that in PykA_PA_ and PykF_EC_, PykF_PA_ contained an additional structure denoted Cα1′ (Figure 4). Cα1′ is a short helix spanning Tyr338–Glu343 and precedes the longer Cα1 helix (Figure 4 and Supplementary Figure 4). Cα1′ is important because it is integrally associated with the A-A interface.

Amino acid sequence analysis shows that the active site of PykF_PA_ is comprised of strictly conserved residues (Supplementary Figure 4). Superposition of these active site residues in PykF_PA_ (no bound substrate, PDB 7OO1), PykA_PA_ (bound to a substrate analog, malonate-Mg^2+^, PDB 6QXL), and PykF_EC_ (no bound substrate, PDB 1PKY) revealed that the constellation of the active site residues in PykF_PA_ (comprised of the side chains from Arg35, Lys217, Glu219, Gly242, Asp243, and Thr275) adopts a similar configuration in the active (holo) structure, represented by PDB:6QXL, and in the inactive (apo) structure, represented by PDB:1PKY (Supplementary Figure 6). The only residue in the active site constellation of PykA_PA_ that can be said to adopt a significantly different configuration between the inactive and active structures is Asp243 in PykF_PA_. In the substrate bound (active) structure, 6QXL, this residue re-orients away from the active site slightly in order to make space for the Mg^2+^ that is chelated by the substrate (Supplementary Figure 6).

**FIGURE 5:**
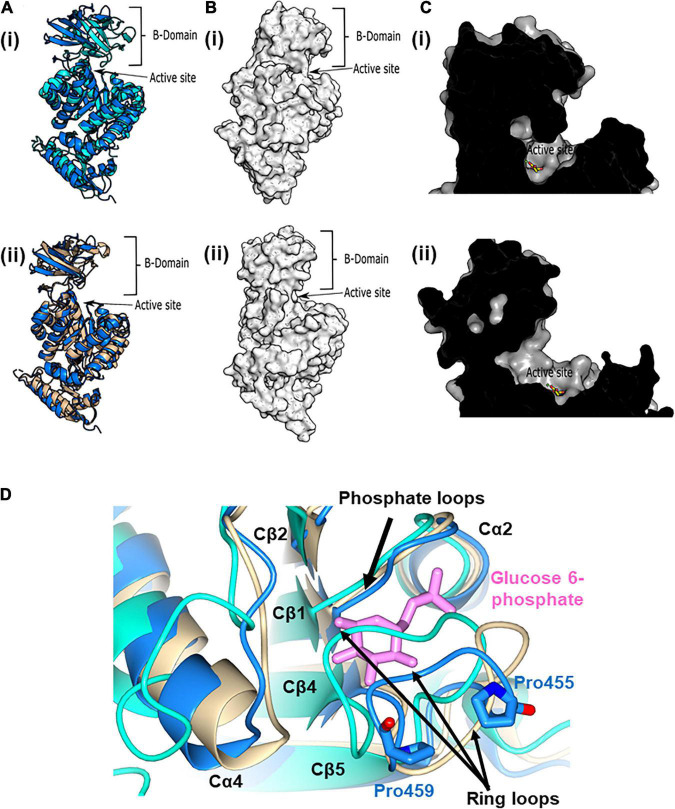
The active and allosteric sites of PykF. **(A)** (i) Cartoon representation of the crystal structure of a presumed inactive conformation of pyruvate kinase (PykF_PA_; 7OO1, blue) overlain onto the crystal structure of a presumed active configuration of the enzyme (PykA_PA_; 6QXL, cyan). (ii) Cartoon representation of the crystal structure of PykF_PA_ (inactive configuration, 7OO1, blue) overlain onto the crystal structure of PykF_EC_ (inactive configuration, 1PKY, yellow). **(B)** Surface representation of the crystal structure of PykA_PA_ [(i) 6QXL] and PykF_PA_ [(ii) 7OO1, blue]. **(C)** (i) Cross-section through the active site of PykA_PA_ (active configuration of the enzyme, 6QXL) The substrate analog, malonate, is shown in yellow. (ii) Cross-section through the active site of PykF_PA_ (inactive configuration of the enzyme, 7OO1). The superimposed binding mode of the malonate from 6QXL is shown in yellow. **(D)** Partial closure of the allosteric site of PykF_PA_. Superposition of the allosteric site in PykF_PA_ (7OO1, blue), PykF_EC_ (1PKY, yellow), and PykA_PA_ (6QXL, cyan) showing disposition of the ring loop of PykF_PA_ toward the allosteric site, probably determined by the configuration of Pro455 and Pro459. The G6P bound to PykA_PA_ is shown as pink sticks.

In spite of the relatively small differences in the active site configuration between the active and inactive conformers of pyruvate kinase, activation resulted in a large shift in the position of the B-domain relative to the A-domain (Figures 5A,B). In the active (substrate bound) configuration, the B-domain clearly rotates toward the A-domain. This rotation causes a partial closing of the entrance to the active site compared with the inactive (no substrate, pdb:7OO1 and 1PKY) configurations (Figure 5C). The inactive configuration of the PykF_PA_ active site was further confirmed by analysis of the orientation of the terminal arginine residue (Arg289) in helix Aα7 (Supplementary Figure 6). In inactive structures such as PykF_EC_ PDB 1PKY, Arg292 (equivalent to Arg289 in PykF_PA_) interacts with Asp297 (equivalent to Asp294 in PykF_PA_) from the adjacent protomer, forming an Aα7-Aα7 bond. This interaction is preserved in the PykF_PA_ structure presented here. However, in the structure of active PykA_PA_ (PDB 6QXL) Arg296 (equivalent to Arg289 in PykF_PA_) reorients toward the active site helix (Aα6′), forming an Aα7-Aα6′ interaction.

In the previously solved structure of G6P-bound PykA_PA_, the phosphate and sugar ring moieties of G6P are anchored in the allosteric pocket *via* a “phosphate loop” (Cβ1-Cα2 loop) and a “ring loop” (Cβ4-Cβ5 loop), respectively (Abdelhamid et al., 2019). Indeed, closure of the allosteric site by the ring loop has been proposed to accompany binding of G6P (Zhong et al., 2017; Abdelhamid et al., 2019). Surprisingly, superposition of PykF_PA_ (empty allosteric site), PykA_PA_ (PDB 6QXL, G6P bound to allosteric site), and PykF_EC_ (PDB 1PKY, empty allosteric site) revealed that the ring loop in PykF_PA_ adopts an intermediate conformation between the fully “open” configuration seen in PykF_EC_ and the “closed” configuration in PykA_PA_ (Figure 5D). This partially closed configuration of the ring loop in apo-PykF_PA_ likely reflects the nature of the amino acids comprising the ring loop (residues 454–461).

### Site-Directed Mutagenesis of the Ring Loop in PykF_PA_

Compared with PykA_PA_ and PykF_EC_, the ring loop in PykF_PA_ is unusual because it is flanked by two proline residues, Pro455 and Pro459 (Figure 5D and Supplementary Figure 4). Although Pro455 is conserved in several pyruvate kinases (Supplementary Figure 4), Pro459 is not conserved. Proline residues can impact on the conformational freedom of adjacent amino acids, so we wondered whether these flanking prolines may alter the flexibility of the loop and perhaps play an important role in dictating the response of the protein to the binding of allosteric regulators [In PykA, structural data indicate that G6P binding pulls the ring loop across the effector binding site, which, in turn, displaces a mobile loop at the end of Cα4. The consequent conformational change is transmitted through the enzyme to the A-A promoter interface, and thence to the active site (Abdelhamid et al., 2019)]. To investigate this further, we used site-directed mutagenesis to mutate Pro455 and Pro459 (separately) to alanine and examined the impact of these changes on the kinetics of the enzyme and its response to G6P. The Pro455Ala mutation increased the activity of the enzyme, with a low S_0_._5_ value and Michaelis-Menten (hyperbolic) kinetics irrespective of the presence or absence of G6P, suggesting that enzyme is likely in a more active conformation (Table 1 and Supplementary Figure 7A). By contrast, the Pro459Ala mutation did the opposite, decreasing the activity of the enzyme and favoring a conformation that was only poorly responsive to G6P. The Pro459Ala mutant protein exhibited strongly sigmoidal kinetics in the presence and absence of the regulator (Table 1 and Supplementary Figure 7B). Given the contrasting effects of the Pro→Ala substitutions at each end of the ring loop, we next wondered what would happen if both residues were replaced with Ala in the same protein. The Pro455Ala/Pro459Ala double mutant had a high S_0_._5__(PEP)_ (1.9–2.0 mM) and displayed essentially no cooperativity (the Hill coefficient, h ≈ 1), irrespective of the presence of G6P (Table 1 and Supplementary Figure 7C). Taken together, these data indicate that the specific nature of the residues flanking the ring loop has a profound impact on the ability of the enzyme to respond to allosteric regulators.

### Inter-Protomer Interactions in PykF_PA_

The A-A interface in PykF_PA_ was formed between the adjacent A-domains from chain A and chain B, and from chain C and D, whereas the C-C interface was present between the adjacent C-domains of chain A and chain C, and of chain B and D (Figure 4). Interactions across the A-A and C-C inter-protomer spaces of PykF_PA_ are listed in Supplementary Table 2.

**FIGURE 6:**
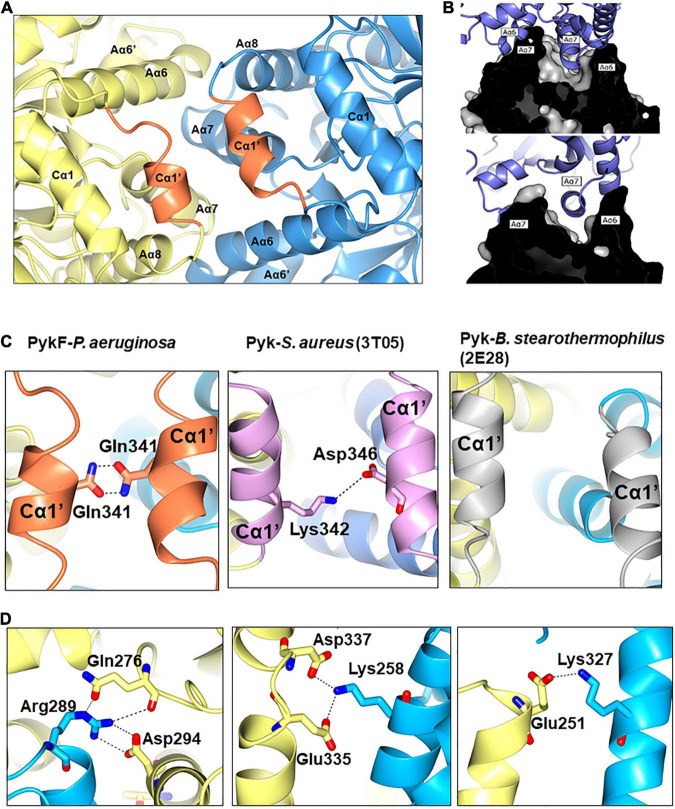
The A-A interface of PykF. **(A)** Secondary structures present at the A-A interface in PykF. The Cα1′ helices are shown in coral. **(B)** The interlocking of α-helices Aα6 and Aα7 at the A-A interface (upper and lower panels show a cross-section through the interface at different tilt angles). **(C)** Close-up view of the interactions at the Cα1′-Cα1′ interspace in bacterial species that have a Cα1′-like structure. **(D)** Close-up view of the A-A interface in PykF showing salt bridge formation across the interface. Of note, the A-A interface of *E. coli* PykF does not contain a Cα1′-like structure or salt bridges.

The A-A interface of PykF_PA_ comprises Aα6, Aα7, Aα8, Cα1′, the Aα7-Aβ7 loop and the Aα8-Cα1′ loop (Figure 6). From analysis of the structure, it appears that the A-A interaction is dominated by the interlocking of Aα6 and Aα7. The Aα7 helix from the first A domain sits in the groove that is formed between Aα6 and Aα7 in the second A domain, with the first residue of Aα7 (Arg289) penetrating deep into the groove and forming a hydrogen bond with Asp294 (Figure 6D). Interestingly, the orientation of Arg289 correlates well with the activation state of the protein, and in PykF_PA_, adopts a configuration commensurate with the inactive state (Supplementary Figure 6B). The A-A interface is also further stabilized *via* a web of additional potential hydrogen bonds and salt bridges (Lys258-Asp337/Glu335, and Glu251-Lys327) (Figure 6D and Supplementary Table 2). The Cα1′ helix, which is absent from PykA_PA_ and PykF_EC_, connects the opposing A-domains of PykF_PA_ through a Cα1′-Cα1′ interaction (Figures 6A,C). As shown in Table 3, the PK isozymes from *Bacillus stearothermophilus* and *Staphylococcus aureus* also contain Cα1′-like helices, similar to PykF_PA_. In PykF_PA_, the Cα1′-Cα1′ interaction is mediated by the side chains of Gln341 from each protomer, which form a glutamine dimer linked by a probable pair of reciprocal hydrogen bonds (3 Å). By contrast, in the *S. aureus* enzyme the Cα1′-Cα1′ interaction is mediated by a salt bridge, whereas in the PK from *B. stearothermophilus* there is no obvious Cα1′-Cα1′ interaction (Figure 6C). To test whether the putative Gln341-Gln341 hydrogen bond(s) might play a role in the conformational transitions associated with PykF_PA_ function, we mutated this residue to alanine. However, this had little discernible effect on the kinetics of the enzyme in the presence or absence of G6P (Supplementary Figure 7D).

**FIGURE 7:**
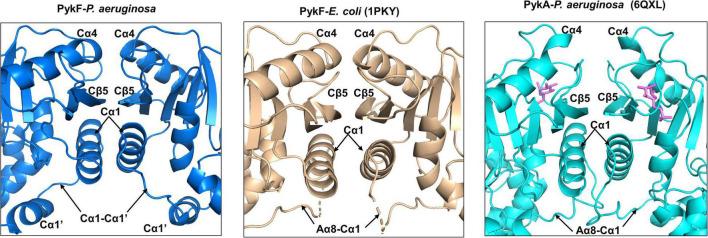
Secondary structures of the C-C interface of PykF_PA_ (blue), PykF_EC_ (wheat) and PykA_PA_ (cyan). The diagram shows that the C-C interface in PykF_PA_ adopts almost the same configuration as the C-C interface in PykF_EC_, likely due to the absence of bound regulator in the allosteric site. The G6P bound to PykA_PA_ is shown as pink sticks.

The secondary structures at the C-C interface in PykF_PA_ were similar to those in PykF_EC_ (PDB 1PKY, open allosteric site) (Figure 7). The interface is comprised of the α-helices Cα4 and Cα1, which flank Cβ5. The interface is more planar than the A-A interface with no binding pockets or grooves. At the C-C interface of PykF_PA_ and PykF_EC_, Cα4 from one protomer abuts Cα4 of the adjacent protomer, and Cβ5 forms an extended β-sheet with the respective Cβ5 of the adjacent protomer. Additionally, and unlike PykA_PA_, Cα1 and the Cα1-Cα1′ loop (equivalent to loop Aα8-Cα1 in PykF_EC_) from each protomer are not intimately associated in these enzymes, but instead, exhibit only a weak hydrophobic interaction between the helices (Figure 7). This configuration at the C-C interface is indicative of an empty allosteric site, as these interactions are re-arranged in G6P-bound PykA_PA_ (Figure 7). In PykA_PA_, G6P binding displaces Cα4 concomitantly bringing Cα1 and the Aα8-Cα1 loop closer to the interface (Abdelhamid et al., 2019). Interestingly, and despite its superficial resemblance to the C-C interface in PykF_EC_, the C-C interface of PykF_PA_ contains a number of bonds between non-conserved residues (Supplementary Figure 4).

**FIGURE 8:**
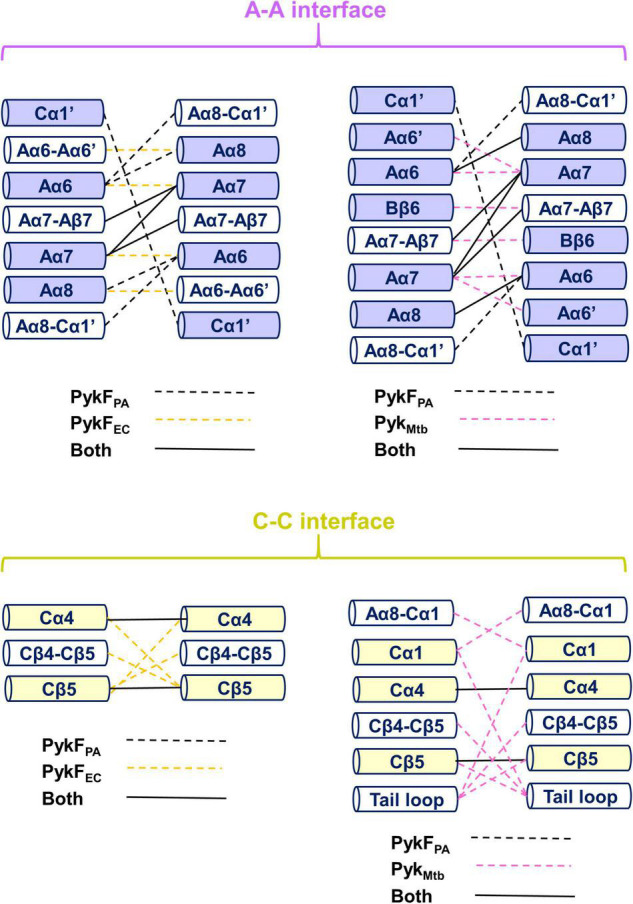
The inter-protomer interfaces in PykF. The top diagram shows a comparison between the A-A interface in PykF_PA_, and (left) PykF_EC_ and (right) Pyk_Mtb_. The bottom panel shows a comparison between the C-C interface in PykF_PA_ and (left) PykF_EC_ and (right) Pyk_Mtb_. Interactions unique to PykF_PA_ (black dashed lines), PykF_EC_ (yellow dashed lines), and Pyk_Mtb_ (pink dashed lines) are shown. Interactions present in PykF_PA_ and any of the two structures are shown as solid black lines.

Investigation of inter-protomer interactions can potentially provide insight into the mechanism by which conformational signals are transmitted between the different protomers (Wooll et al., 2001). However, comparison of the inter-protomer interactions between secondary structural elements in the apo structures of PykF_PA_ (PDB 7OO1), PykF_EC_ (PDB 1PKY), and Pyk_Mtb_ (PDB 5WRP) revealed that these interactions are not especially well-conserved between the species (Figure 8). The A-A interface in PykF_PA_ is primarily distinguished by bonding of Cα1′ on one protomer with Cα1′ on the other, and by bonding of the Aα8-Cα1′ loop on one protomer with Aα6 on the other (Figure 8). These interactions are not present at the A-A interface of PykF_EC_ or Pyk_Mtb_. On the other hand, both PykF_EC_ and Pyk_Mtb_ exhibit Aα6-Aα7 interactions (absent in PykF_PA_). In PykF_EC_, the Aα6-Aα6′ loop also contributes to the A-A interface–interactions that are again, absent in PykF_PA_. Similarly, the active site helix Aα6′ and parts of the B domain are present at the A-A interface in Pyk_Mtb_, but not in PykF_PA_. Analysis of the C-C interface reveals a similar story. Compared with PykF_EC_, the C-C interface of PykF_PA_ does not include the ring loop (Cβ4-Cβ5). Absence of the ring loop from the interface in PykF_PA_ is likely related to the conformational constraints introduced by the two proline residues (Pro455 and Pro459) that flank the loop in this enzyme. By contrast, the C-C interface of PykF_EC_ does include the ring loop, but without any salt bridges. In Pyk_Mtb_, the C-C interface comprises the ring loop, the tail loop, Aα8-Cα1 loop and the Cα1 helix (all absent from the interface in PykF_PA_). The discrepancy between the inter-protomer spaces of PykF_PA_ (on the one hand) and PykF_EC_ or Pyk_Mtb_ (on the other) suggests that PykF_PA_ most likely depends on a distinctive mechanism for allosteric signal transduction compared with previously proposed models.

## Discussion

In this work, we characterized the second encoded pyruvate kinase isozyme of PA, PykF_PA_. Based on the presumed function (allantoin catabolism) of the ORFs in the *pykF*-containing cluster, we predicted that PykF expression might be stimulated in the presence of allantoin, and this was indeed the case. Allantoin is a diureide derived from uric acid; itself a product of purine degradation. DNA-derived purines can be abundant in the airways of people with cystic fibrosis (CF), which is a common infection niche colonized by *P. aeruginosa* (Kumar et al., 2019) and indeed, these compounds can become abundant enough to support the appearance of auxotrophs defective in purine biosynthesis (Al Mahmud et al., 2021).

But why encode a dedicated second pyruvate kinase isozyme (PykF_PA_) when the organism already contains a similarly bioactive isozyme (PykA_PA_) which appears to be expressed under most conditions? The answer to this question is not yet clear, although we note that during growth on allantoin as a sole carbon source, the *pykF* mutant displayed a growth defect relative to the *pykA* mutant (Supplementary Figure 1), which strongly suggests that the two isozymes are not equivalent. One possibility is that one or more of the intermediates that are generated during growth on allantoin may differentially inhibit PykA_PA_ but not PykF_PA_. One obvious candidate in this regard would be hydroxypyruvate (derived from the spontaneous non-enzymatic isomerization of tartronate semialdehyde–the predicted product of the glyoxylate carboligase-catalyzed reaction). However, hydroxypyruvate had no significant differential effect on the activity of PykA_PA_ and PykF_PA_ (*data now shown*), so we infer that some other intermediate is likely responsible. If the function of PykF_PA_ is indeed to compensate for the inhibition of PykA_PA_ during growth on allantoin, this would also explain why the two enzymes have very similar regulatory properties.

The *pykF* ORF encodes a functional pyruvate kinase with kinetic parameters roughly comparable with those of PykA_PA_. Purified PykF_PA_ and PykA_PA_ were also activated by a broadly similar set of allosteric regulators. Notably, and although the PykF sub-family of PK isozymes were originally functionally designated as being regulated by fructose 1,6-*bis*phosphate, this metabolite had little impact on the activity of PykF_PA_. Similarly, whereas the PykA family of isozymes were originally designated thus because they are regulated by AMP, this molecule had no impact on the activity of PykA_PA_ (Abdelhamid et al., 2019) although it is a moderate activator of PykF_PA_. These data may suggest that assignation of a PK isozyme into the “PykA” or “PykF” sub-family, which nowadays, is largely based on sequence analyses, is a convenience that does not necessarily have any associated functional significance. However, the insensitivity of PykF_PA_ (and also PykA_PA_) to fructose 1,6-*bis*phosphate may also have a structural explanation. Analysis of the fructose 1,6-*bis*phosphate-activated yeast PK revealed that the negatively charged phosphate moiety at the 1 position of the sugar ring in the regulator molecule interacts with a positively charged residue (arginine) located on the nearby Cα4 (Jurica et al., 1998). Other fructose 1,6-*bis*phosphate-activated PykF isoforms also contain candidate positively charged residue(s) on Cα4 at the equivalent position (Supplementary Figure 8). By contrast, the Cα4 (residues 425–438) of PykF_PA_ lacks a positively charged residue at this position on Cα4, likely accounting for its insensitivity to fructose 1,6-*bis*phosphate. This insensitivity to fructose 1,6-*bis*phosphate makes good physiological sense, since, due to the absence of phosphofructokinase (and thus, a conventional EMP pathway) in PA, the only time this intermediate will accumulate is during gluconeogenesis. Clearly, it would be wasteful to stimulate EDP glycolysis in these circumstances.

One of the main differences between the structure of PykF_PA_ and the pyruvate kinases from other Proteobacteria is that PykF_PA_ contains a Cα1′ helix. In many species, Cα1′ is replaced by a long uninterrupted loop (the Aα8-Cα1 loop) connecting the A- and C-domains (Supplementary Figure 9). Some Firmicutes also have a Cα1′-like structure (Figure 6C), although with a diverse amino acid sequence (Supplementary Figure 4). This indicates that the function of Cα1′ is likely species-specific. In PykA_PA_, the Aα8-Cα1 loop has been implicated in transmission of the conformational signal from the allosteric site to the active site (Abdelhamid et al., 2019), so Cα1′ is located at a strategically important site in the enzyme (Figures 6, 7). However, abolition of the presumed reciprocal H-bonds between the side chain of residue Gln341 on each protomer (H-bonds which apparently stabilize Cα1′-Cα1′ interactions between protomers) had little impact on the activity or G6P-dependent regulation of the enzyme, so the functional role(s), if any, of this secondary structure remain unclear.

Analysis of the C-C interface in apo-PykF_PA_ shows that the ring loop between Cβ4 and Cβ5 is not a part of the interface. This contrasts with the same structure in apo-PykF_EC_ and in the pyruvate kinase from *M. tuberculosis* (Pyk_Mtb_). This is apparently due to the partial retraction of the ring loop from the interface and its movement toward the allosteric site, a feature that is presumably attributable to the presence of Pro459 (Figure 5C). A possible role for the other ring loop-flanking proline, Pro455, in this is made less likely by the fact that whereas PykF_Ec_ lacks a proline at the equivalent position, Pyk_Mtb_ retains one. Nevertheless, the conformational importance of Pro455 is confirmed by the fact that the mutant Pro455Ala PykF_PA_ protein is locked into an essentially constitutively active configuration. By contrast, mutation of Pro459 to Ala apparently locked the enzyme into a non-activatable (by G6P) state. We speculate that in this mutant protein, the ring loop engages in other interactions that prevent it from fully folding over the G6P-binding site following interaction with the ligand, thereby blocking the downstream conformational transitions leading to activation.

The A-A inter-protomer space of PykF_PA_ is distinguished from that in PykF_EC_ and Pyk_Mtb_ by the presence of the Cα1′ helices, and by bonding of the Aα8-Cα1′ loop with Aα6 (Figure 8). In light of this, it is tempting to extrapolate a general mechanism that may explain the allosteric regulation of PykF_PA_. Upon binding of a regulator molecule in the allosteric pocket of PykF_PA_, the ring loop folds down across the bound ligand, along similar lines to what is observed in G6P-bound PykA_PA_ (Zhong et al., 2017; Abdelhamid et al., 2019). This movement of the ring loop would be expected to induce rearrangement of the structures at the C-C interface, including breaking of Cα4-Cα4 interactions and building of a Cα1-Cα1 interaction. These proposed changes at the C-C interface are common in the allosteric regulation of many bacterial and non-bacterial pyruvate kinases (Mattevi et al., 1996; Jurica et al., 1998; Abdelhamid et al., 2019) and naturally lead to a set of inferred downstream changes in which the shifting of Cα1 toward the C-C interface “pulls” on the Cα1-Cα1′ loop, Cα1′ helix, Aα8-Cα1′ loop and/or Aα8 helix. These movements of the Aα8-Cα1′ loop and/or Aα8 away from the A-A interface would free Aα6 and the Aα6′ active site helix to move (accounting for the change in relative orientation of the A- and B-domains on the protein, leading to closure of the active site) and to promote new interactions at the A-A interface. Such a set of proposed conformational changes would provide a direct structural pathway linking events at the ligand (effector) binding site and the active site. Consistent with this model, recruitment of a Cα1′-like helix to the C-C interface has been observed before upon occupation of the allosteric site in a yeast pyruvate kinase by a regulator (Jurica et al., 1998). However, these proposed changes do not account for the extreme apparent “locked on” and “locked off” phenotypes of the ring loop Pro→Ala mutants. They also fail to take into account the fact that the most potent regulators of PykF_PA_ are PPP sugars with linear (not ring-like) configurations, which may or may not bind to the inferred G6P-binding site on the protein; an issue that we are currently investigating.

In summary, we present here the structure, function and regulation of a second pyruvate kinase isoform, PykF_PA_, from *P. aeruginosa*. Unlike the PykF_Ec_ and PykA_Ec_ isoforms in *E. coli*, which carry out essentially the same “metabolic job” but under different conditions of oxygen availability (Zhao et al., 2017), in *P. aeruginosa*, it is clear that PykA_PA_ is “the main” pyruvate kinase employed under most growth conditions, and that PykF_PA_ has a more dedicated role in allantoin degradation. Crucially, our structural and mechanistic data indicate that the specific nature of the “ring loop” interactions around the presumed G6P-binding site in PykF_PA_ introduce a hitherto unexpected layer of complexity into our understanding of how allosteric transitions are accomplished. Indeed, our future efforts are aimed at trying to obtain the crystal structure of the “locked on” and “locked off” conformers, and in examining how the more potent (than G6P) PykF_PA_ and PykA_PA_ allosteric regulators work.

## Materials and Methods

### PykF Expression

PW8308 (a Tn:*pykA* mutant) and PW3705 (a Tn:*pykF* mutant) were obtained from the UWGC *P. aeruginosa* mutant bank. The Tn insertion in each mutant has been previously confirmed (Abdelhamid et al., 2019). A single colony of each relevant strain (wild-type PAO1, the *pykA* mutant, and the *pykF* mutant) was picked and used to inoculate 10 mL LB. The cultures were grown overnight at 37°C on a rotating drum. The cells were then pelleted (3200 × *g*, 20°C, 5 min) and washed three times in 10 mL sterile PBS. The cells were then inoculated 200 mL M9 minimal media containing either glucose (14 mM) or allantoin (21 mM) or a combination of both carbon sources, to an initial OD_600_ of 0.05. These concentrations of each carbon source were chosen because they contain the same molar number of carbon atoms. The cultures were incubated for 24 h in orbital shaker at 37°C with good aeration (200 rpm). The cells were then pelleted by sedimentation (3200 × *g*, 4°C, 10 min) and resuspended in 2 mL lysis buffer [comprising 50 mM Tris–HCl (pH 7.5), 400 mM NaCl, 10 mM imidazole, 5% (v/v) glycerol and one EDTA-free protease inhibitor cocktail per 50 mL buffer]. The samples were sonicated on ice to completion and clarified by centrifugation (14,600 × *g*, 4°C, 5 min). The protein concentration in the clarified extract was quantified using the Bradford Assay (BSA standard). Samples (20 μg protein per lane) were then denatured in SDS sample buffer and resolved by SDS-PAGE (9% polyacrylamide gels). Following PAGE, the proteins were transferred to immobilon-FL PVDF membranes (Merck Millipore) using a Bio-Rad *Trans*-Blot Turbo (mixed MW program; 2.5 A, 7 min for 2 mini gels). The membranes were washed 3× for 5 min in phosphate-buffered saline containing 0.1% (v/v) TWEEN-20 and blocked overnight in the same buffer containing 5% w/v skimmed milk. The primary antibody [anti-PykA (1:2000) or anti-PykF (1:3000) (Abdelhamid et al., 2019)] was incubated with each membrane for 60 min at room temperature. The membranes were then washed 3× for 5 min in PBS-TWEEN wash buffer before addition of the secondary antibody [IRDye 800CW Goat anti-Rabbit (LI-COR), 1:15,000]. After 60 min, the membranes were washed 3× in PBS-TWEEN and imaged using a ChemiDoc MP Imaging System (Bio-Rad).

### Cloning, Overexpression and Purification of PykF

PykF was over-expressed in *E. coli* strain BL21 (DE3) containing plasmid pET-19m (*pykF*) and purified as previously described (Abdelhamid et al., 2019).

### Kinetic Analysis of Purified PykF

Pyruvate kinase activity was measured using a lactate dehydrogenase (LDH)-coupled assay following our previous protocol for purified PykA (Abdelhamid et al., 2019) except that unless otherwise stated, purified PykF was added to a final concentration of 0.25 μg/mL to start the reactions. Regulator screens were also carried out as previously described (Abdelhamid et al., 2019). In all experiments, regulators were added at 1 mM final concentration except for R5P, X5P, and RL5P which were used at 0.15, 0.5, and 0.5 mM, respectively. GraphPad prism 7 was used to analyze the data and to extract the kinetic constants. All experiments were carried out in triplicate. Raw and processed kinetic data are provided in Supplementary Tables 3, 4.

### Analytical Ultracentrifugation

Analytical ultracentrifugation analyses were carried out as previously described (Abdelhamid et al., 2019). Data analysis and calculations of buffer viscosity, protein partial specific volumes and frictional rations were done using SEDFIT (Schuck, 2000) and SEDNTERP (Hayes et al., 1995).

### Crystallization of PykF

PykF was crystallized using the sitting drop vapor diffusion method. MRC 2-drop plates (Swissci) were used and solutions were dispensed using the Mosquito robotics system (SPT Labtech). Purified PykF [29 mg/mL in 20 mM Tris–HCl, 100 mM NaCl, 5% (v/v) glycerol, 1 mM DTT, 0.1 mM EDTA, 20 mM MgCl_2_, 200 mM KCl, 2 mM PEP (pH 7.5)] was mixed 1:1 (200 nL each) with the reservoir buffer containing 25% (w/v) PEG6000 and 0.1 M Hepes (pH 7.5). Crystals grew within 1 week. The crystals were mounted on nylon loops and cryoprotected in mother liquor supplemented with 40% (v/v) glycerol before being flash frozen in liquid N_2_.

### X-ray Diffraction, Structure Determination and Refinement

Diffraction data were collected at the Diamond Light Source (Didcot, United Kingdom) on beamline IO4-1 (MX14043-47). The PykF structure was obtained by molecular replacement using Phaser MR (McCoy et al., 2007) and a PykF ensemble generated by the Swiss model (Waterhouse et al., 2018) as a structural template. Coot (Emsley et al., 2010) was used for model building, and refinement was carried out using Phenix.refine (Adams et al., 2010). The overall model of PykF was acceptable except that the electron density signal was weak at the Cβ3 strand (now modeled as a loop) and the three terminal residues (now unmodelled) of chain A. The structural coordinates of PykF were deposited in the PDB with the accession code 7OO1. PDBePISA (Krissinel and Henrick, 2007) was used for analysis of the tetramer and ligand interfaces. Figures were generated using CCP4mg (McNicholas et al., 2011).

### Amino Acid Sequence Analysis

Amino acid sequences were extracted from UniProt in FASTA format, aligned by Clustal Omega (Goujon et al., 2010; Sievers et al., 2014) and formatted for display using ESPript (Robert and Gouet, 2014).

### Site-Directed Mutagenesis

Site-directed mutagenesis (Q341A, P455A, and P459A mutants) was carried out by overlap extension PCR using pET-19m (*pykF*) as a template. The *pykF* cloning primers and an extra pair of overlap primers were designed and used for site-directed mutagenesis (Supplementary Table 1). Briefly, in the first PCR step, the 5′ region of the *pykF* gene was amplified using the *pykF* forward cloning primer and the corresponding reverse overlap primer, and the 3′ region was amplified using the *pykF* reverse cloning primer and relevant forward overlap primer. The purified 5′ and 3′ region PCR products were subsequently mixed and used as a template for PCR -amplification using the *pykF* cloning primers. The resulting PCR product was ligated to pET-19m using T4 DNA ligase (NEB). Each mutation was confirmed by DNA sequencing. The expression and purification of the mutated PykF proteins was the same as for the wild-type protein (Abdelhamid et al., 2019).

## Data Availability Statement

The datasets presented in this study can be found in online repositories. The names of the repository/repositories and accession number(s) can be found below: http://www.wwpdb.org/, PDB 7OO1.

## Author Contributions

YA carried the kinetic and regulatory analysis of the wild-type protein, obtained the crystallographic data, and drafted the manuscript. MWa carried out the site-directed mutagenesis and subsequent characterization of the mutant proteins. SP carried out the expression analyses. PB assisted in phasing and solving the crystal structure. MWe conceived of the study, analyzed the data, and assisted in preparation of the manuscript. XC and TR assisted with the structural analysis. All authors contributed to the article and approved the submitted version.

## Conflict of Interest

The authors declare that the research was conducted in the absence of any commercial or financial relationships that could be construed as a potential conflict of interest.

## Publisher’s Note

All claims expressed in this article are solely those of the authors and do not necessarily represent those of their affiliated organizations, or those of the publisher, the editors and the reviewers. Any product that may be evaluated in this article, or claim that may be made by its manufacturer, is not guaranteed or endorsed by the publisher.

## References

[B1] Abdelhamid Y. Brear P. Greenhalgh J. Chee X. Rahman T. Welch M. (2019). Evolutionary plasticity in the allosteric regulator binding site of pyruvate kinase isoform PykA from *Pseudomonas aeruginosa*. *J. Biol. Chem.* 294 15505–15516. 10.1074/jbc.RA119.009156 31484721 PMC6802521

[B2] Adams P. D. Afonine P. V. Bunkóczi G. Chen V. B. Davis I. W. Echols N. (2010). PHENIX: a comprehensive Python-based system for macromolecular structure solution. *Acta Crystallogr. Sect. D Biol. Crystallogr.* 66 213–221. 10.1107/S0907444909052925 20124702 PMC2815670

[B3] Al Mahmud H. Baishya J. Wakeman C. A. (2021). interspecies metabolic complementation in cystic fibrosis pathogens via purine exchange. *Pathogens* 10:146. 10.3390/pathogens10020146 33535659 PMC7912780

[B4] Al-Zaid Siddiquee K. Arauzo-Bravo M. J. Shimizu K. (2004). Metabolic flux analysis of pykF gene knockout *Escherichia coli* based on 13C-labeling experiments together with measurements of enzyme activities and intracellular metabolite concentrations. *Appl. Microbiol. Biotechnol.* 63 407–417. 10.1007/s00253-003-1357-9 12802531

[B5] Baek Y. H. Nowak T. (1982). Kinetic evidence for a dual cation role for muscle pyruvate kinase. *Arch. Biochem. Biophys.* 217 491–497. 10.1016/0003-9861(82)90529-X 7138020

[B6] Bücker R. Heroven A. K. Becker J. Dersch P. Wittmann C. (2014). The pyruvate-tricarboxylic acid cycle node: a focal point of virulence control in the enteric pathogen Yersinia pseudotuberculosis. *J. Biol. Chem.* 289 30114–30132. 10.1074/jbc.M114.581348 25164818 PMC4208018

[B7] Bumann D. Schothorst J. (2017). Intracellular *Salmonella* metabolism. *Cell. Microbiol.* 19:e12766. 10.1111/cmi.12766 28672057

[B8] Cusa E. Obradors N. Baldomà L. Badía J. Aguilar J. (1999). Genetic analysis of a chromosomal region containing genes required for assimilation of allantoin nitrogen and linked glyoxylate metabolism in *Escherichia coli*. *J. Bacteriol.* 181 7479–7484. 10.1128/jb.181.24.7479-7484.1999 10601204 PMC94204

[B9] Drechsler E. R. Boyer P. D. Kowalsky A. G. (1959). The catalytic activity of carboxypeptidase-degraded aldolase. *J. Biol. Chem.* 234 2627–2634. 13818028

[B10] Emsley P. Lohkamp B. Scott W. G. Cowtan K. (2010). Features and development of Coot. *Acta Cryst.* 66 486–501. 10.1107/S0907444910007493 20383002 PMC2852313

[B11] Garcia-Olalla C. Garrido-Pertierra A. (1987). Purification and kinetic properties of pyruvate kinase isoenzymes of *Salmonella* typhimurium. *Biochem. J.* 241 573–581. 10.1042/BJ2410573 3297035 PMC1147599

[B12] Goujon M. McWilliam H. Li W. Valentin F. Squizzato S. Paern J. (2010). A new bioinformatics analysis tools framework at EMBL-EBI. *Nucleic Acids Res.* 38 W695–W699. 10.1093/nar/gkq313 20439314 PMC2896090

[B13] Hayes D. Laue T. Philo J. (1995). *Program Sednterp: Sedimentation Interpretation Program.* Thousand Oaks, CA: Alliance Protein Laboratories.

[B14] Hofmann J. Heider C. Li W. Krausze J. Roessle M. Wilharm G. (2013). Recombinant production of Yersinia enterocolitica pyruvate kinase isoenzymes PykA and PykF. *Protein Expr. Purif.* 88 243–247. 10.1016/j.pep.2013.01.010 23384479

[B15] Jurica M. S. Mesecar A. Heath P. J. Shi W. Nowak T. Stoddard B. L. (1998). The allosteric regulation of pyruvate kinase by fructose-1,6-bisphosphate. *Structure* 6 195–210. 9519410 10.1016/s0969-2126(98)00021-5

[B16] Kachmar J. Boyer P. (1953). Kinetic analysis of enzyme reactions. II. The potassium activation and calcium inhibition of pyruvic phosphoferase. *J. Biol. Chem.* 200 669–682. 13034826

[B17] Kayne F. J. (1973). 11 Pyruvate Kinase. *Enzymes* 8 353–382. 10.1016/S1874-6047(08)60071-2

[B18] Kersters K. De Ley J. (1968). The occurrence of the Entner-Doudoroff pathway in bacteria. *Antonie Leeuwenhoek* 34 393–408. 10.1007/BF02046462 5304016

[B19] Kohlstedt M. Wittmann C. (2019). GC-MS-based 13 C metabolic fl ux analysis resolves the parallel and cyclic glucose metabolism of *Pseudomonas* putida KT2440 and *Pseudomonas aeruginosa* PAO1. *Metab. Eng.* 54 35–53. 10.1016/j.ymben.2019.01.008 30831266

[B20] Kovachevich R. Wood W. A. (1955a). Carbohydrate metabolism by *Pseudomonas* fluorescens. III. Purification and properties of a 6-phosphogluconate dehydrase. *J. Biol. Chem.* 213 745–756. 10.1016/s0021-9258(18)98206-2 14367335

[B21] Kovachevich R. Wood W. A. (1955b). Carbohydrate metabolism by *Pseudomonas* fluorescens. IV. Purification and properties of 2-keto-3-deoxy-6-phosphogluconate aldolase. *J. Biol. Chem.* 213 757–767. 14367336

[B22] Krissinel E. Henrick K. (2007). Inference of macromolecular assemblies from crystalline state. *J. Mol. Biol.* 372 774–797. 10.1016/j.jmb.2007.05.022 17681537

[B23] Kumar S. S. Penesyan A. Elbourne L. D. H. Gillings M. R. Paulsen I. T. (2019). Catabolism of nucleic acids by a cystic fibrosis *Pseudomonas aeruginosa* isolate: an adaptive pathway to cystic fibrosis sputum environment. *Front. Microbiol.* 10:1199. 10.3389/fmicb.2019.01199 31214142 PMC6555301

[B24] Laughlin L. T. Reed G. H. (1997). The monovalent cation requirement of rabbit muscle pyruvate kinase is eliminated by substitution of lysine for glutamate 117. *Arch. Biochem. Biophys.* 348 262–267. 10.1006/abbi.1997.0448 9434737

[B25] Lessie T. G. Phibbs P. V. (1984). Alternative pathways of carbohydrate utilization in pseudomonads. *Annu. Rev. Microbiol.* 38 359–388. 10.1146/annurev.mi.38.100184.002043 6388497

[B26] Martínez-Solano L. Macia M. D. Fajardo A. Oliver A. Martinez J. L. (2008). Chronic *Pseudomonas aeruginosa* infection in chronic obstructive pulmonary disease. *Clin. Infect. Dis.* 47 1526–1533. 10.1086/593186 18990062

[B27] Mattevi A. Bolognesi M. Valentini G. (1996). The allosteric regulation of pyruvate kinase. *FEBS Lett.* 389 15–19. 8682196 10.1016/0014-5793(96)00462-0

[B28] McCoy A. J. Grosse-Kunstleve R. W. Adams P. D. Winn M. D. Storoni L. C. Read R. J. (2007). Phaser crystallographic software. *J. Appl. Crystallogr.* 40 658–674. 10.1107/S0021889807021206 19461840 PMC2483472

[B29] McNicholas S. Potterton E. Wilson K. S. Noble M. E. M. (2011). Presenting your structures: the CCP4mg molecular-graphics software. *Acta Crystallogr. Sect. D Biol. Crystallogr.* 67 386–394. 10.1107/S0907444911007281 21460457 PMC3069754

[B30] Nikel P. I. Chavarría M. Fuhrer T. Sauer U. de Lorenzo V. (2015). *Pseudomonas putida* KT2440 strain metabolizes glucose through a cycle formed by enzymes of the entner-doudoroff, embden-meyerhof-parnas, and pentose phosphate pathways. *J. Biol. Chem.* 290 25920–25932. 10.1074/jbc.M115.687749 26350459 PMC4646247

[B31] Noy T. Vergnolle O. Hartman T. E. Rhee K. Y. Jacobs W. R. Berney M. (2016). Central role of pyruvate kinase in carbon co-catabolism of mycobacterium tuberculosis. *J. Biol. Chem.* 291 7060–7069. 10.1074/jbc.M115.707430 26858255 PMC4807288

[B32] Oria-Hernández J. Riveros-Rosas H. Ramírez-Sílva L. (2006). Dichotomic phylogenetic tree of the pyruvate kinase family. *J. Biol. Chem.* 281 30717–30724. 10.1074/jbc.M605310200 16905543

[B33] Peekhaus N. Conway T. (1998). What’s for dinner?: entner-doudoroff metabolism in *Escherichia coli*. *J. Bacteriol.* 180 3495–3502. 10.1128/JB.180.14.3495-3502.1998 9657988 PMC107313

[B34] Ponce E. Flores N. Martinez A. Valle F. Bolívar F. (1995). Cloning of the two pyruvate kinase isoenzyme structural genes from *Escherichia coli*: the relative roles of these enzymes in pyruvate biosynthesis. *J. Bacteriol.* 177 5719–5722. 10.1128/JB.177.19.5719-5722.1995 7559366 PMC177388

[B35] Preston M. J. Seed P. C. Toder D. S. Iglewski B. H. Ohman D. E. Gustin J. K. (1997). Contribution of proteases and LasR to the virulence of *Pseudomonas aeruginosa* during corneal infections. *Infect. Immun.* 65 3086–3090. 10.1128/iai.65.8.3086-3090.1997 9234758 PMC175435

[B36] Robert X. Gouet P. (2014). Deciphering key features in protein structures with the new ENDscript server. *Nucleic Acids Res.* 42 W320–W324. 10.1093/nar/gku316 24753421 PMC4086106

[B37] Romano A. H. Conway T. (1996). “Evolution of carbohydrate metabolic pathways,” in *Research in Microbiology*, eds Msadek T. Beloin C. Cabanes D. (Issy-les-Moulineaux: Elsevier Masson SAS), 448–455. 10.1016/0923-2508(96)83998-2 9084754

[B38] Rose I. A. (1970). Stereochemistry of pyruvate kinase, pyruvate carboxylase, and malate enzyme reactions. *J. Biol. Chem.* 245 6052–6056. 5484463

[B39] Schuck P. (2000). Size-distribution analysis of macromolecules by sedimentation velocity ultracentrifugation and lamm equation modeling. *Biophys. J.* 78 1606–1619. 10.1016/S0006-3495(00)76713-0 10692345 PMC1300758

[B40] Seeholzer S. H. Jaworowski A. Rose I. A. (1991). Enolpyruvate: chemical determination as a pyruvate kinase intermediate. *Biochemistry* 30 727–732. 10.1021/bi00217a022 1988060

[B41] Sievers F. Wilm A. Dineen D. Gibson T. J. Karplus K. Li W. (2014). Fast, scalable generation of high-quality protein multiple sequence alignments using Clustal Omega. *Mol. Syst. Biol.* 7 539–539. 10.1038/msb.2011.75 21988835 PMC3261699

[B42] Temple L. M. Sage A. E. Schweizer H. P. Phibbs P. V. (1998). “Carbohydrate catabolism in *Pseudomonas aeruginosa*,” in *Pseudomonas*, ed. Montie T. C. (Boston, MA: Springer), 35–72. 10.1007/978-1-4899-0120-0_2

[B43] Turner K. H. Everett J. Trivedi U. Rumbaugh K. P. Whiteley M. (2014). Requirements for *Pseudomonas aeruginosa* acute burn and chronic surgical wound infection. *PLoS Genet.* 10:e1004518. 10.1371/journal.pgen.1004518 25057820 PMC4109851

[B44] Valentini G. Iadarola P. Somani B. L. Malcovati M. (1979). Two forms of pyruvate kinase in *Escherichia coli* a comparison of chemical and molecular properties. *Biochim. Biophys. Acta Enzymol.* 570 248–258. 10.1016/0005-2744(79)90145-1 387087

[B45] Waterhouse A. Bertoni M. Bienert S. Studer G. Tauriello G. Gumienny R. (2018). SWISS-MODEL: homology modelling of protein structures and complexes. *Nucleic Acids Res.* 46 W296–W303. 10.1093/nar/gky427 29788355 PMC6030848

[B46] Waygood E. B. Sanwal B. D. (1974). The control of pyruvate kinases of *Escherichia coli*. I. Physicochemical and regulatory properties of the enzyme activated by fructose 1,6-diphosphate. *J. Biol. Chem.* 249 265–274. 4588693

[B47] Waygood E. B. Mort J. S. Sanwal B. D. (1976). The control of pyruvate kinase of *Escherichia coli*. Binding of substrate and allosteric effectors to the enzyme activated by fructose 1,6-bisphosphate. *Biochemistry* 15 277–282. 10.1021/bi00647a006 764863

[B48] Waygood E. B. Rayman M. K. Sanwal B. D. (1975). The control of pyruvate kinases of *Escherichia coli*. II. Effectors and regulatory properties of the enzyme activated by ribose 5-phosphate. *Can. J. Biochem.* 53 444–454. 10.1139/o75-061 236081

[B49] Wooll J. O. Friesen R. H. White M. A. Watowich S. J. Fox R. O. Lee J. C. (2001). Structural and functional linkages between subunit interfaces in mammalian pyruvate kinase. *J. Mol. Biol.* 312 525–540. 10.1006/jmbi.2001.4978 11563914

[B50] Zhao C. Lin Z. Dong H. Zhang Y. Li Y. (2017). Reexamination of the physiological role of PykA in *Escherichia coli* revealed that it negatively regulates the intracellular ATP levels under anaerobic conditions. *Appl. Environ. Microbiol.* 83 e316–e317. 10.1128/AEM.00316-17 28363967 PMC5440719

[B51] Zhong W. Cui L. Goh B. C. Cai Q. Ho P. Chionh Y. H. (2017). Allosteric pyruvate kinase-based “logic gate” synergistically senses energy and sugar levels in Mycobacterium tuberculosis. *Nat. Commun.* 8:1986. 10.1038/s41467-017-02086-y 29215013 PMC5719368

