# Supplementary material for: Structure, Function and Regulation of a Second Pyruvate Kinase Isozyme in Pseudomonas aeruginosa
Source: Front Microbiol. 2021 Nov 16;12:790742. doi: 10.3389/fmicb.2021.790742 (PMC8637920; doi:10.3389/fmicb.2021.790742)
Supplement: Supplementary file 1 [file Data_Sheet_1.PDF]

Supplementary Information

Table S1. Bacterial strains, plasmids and primers used in the study.

Primers		Sequence (5'→3')
Cloning primers		
WT	F	GATGAC**C**ATATGACAGCCGACAAGAAAGCCAAGA
	R	CTGAAAGCTTAAGGTCTTTCCCGGATGGATGGAG
Overlap primers		
P455A	F	CCGTGGTGGTCACCGCCGGCGTAGCGTTCGGTCGCCCCGGCTCGACCAACATGC
	R	GCATGTTGGTCGAGCCGGGGCGACCGAACGCTACGCCGGCGGTGACCACCACGG
P459A	F	CACCGCCGGCGTACCGTTCGGTCGCGCCGGCTCGACCAACATGCTGCG
	R	CGCAGCATGTTGGTCGAGCCGGCGCGACCGAACGGTACGCCGGCGGTG
P455A/ P459A	F	GTGGTGGTCACCGCCGGCGTAGCGTTCGGTCGCGCCGGCTCG
	R	CGAGCCGGCGCGACCGAACGCTACGCCGGCGGTGACCACCAC

Table S2. Inter-subunit interactions in $PykF_{PA}$. Each interaction is presented once. Red and black asterisks refer to the formation of salt bridges and hydrogen bonds, respectively. The results are based on interface analysis using PDBePISA. The electron density shown is a $2F_o$-F_c map contoured at 1 σ.

	**Interactions at the A-A interface in PykF**					
**Chain B**	**Secondary structure**	**Distance (Å)**	**Chain A**	**Secondary structure**		
Lys258	Aα6	2.54	Glu335/	loop Aα8-Cα1’	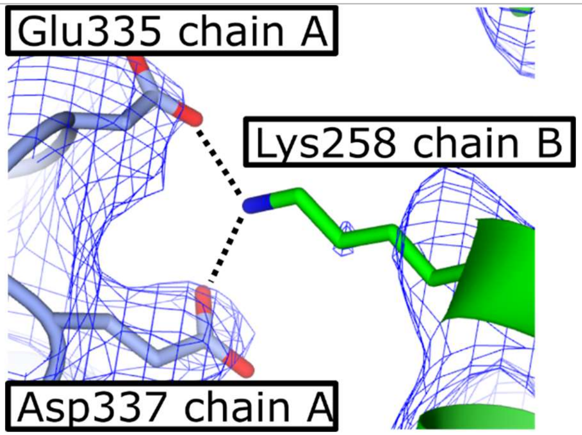	*
Arg289	Aα7	2.68	Gln276	loop Aβ7-Aα7		*

					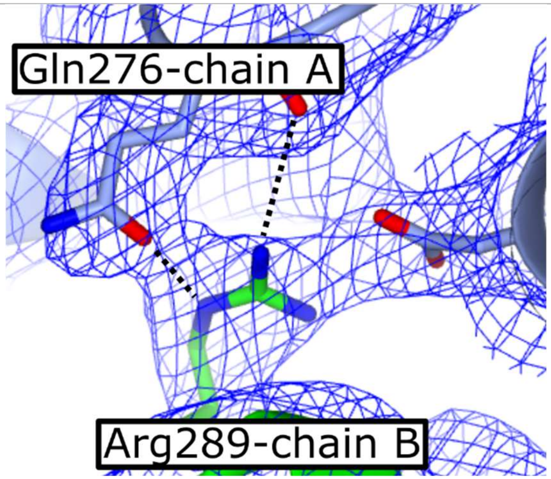	
Arg289	Aα7	2.64	Asp294	Aα7	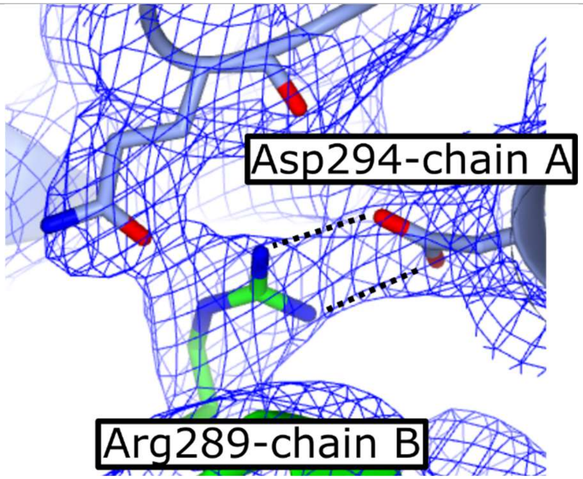	*

Thr297	Aα7	3.84	Thr297	Aα7	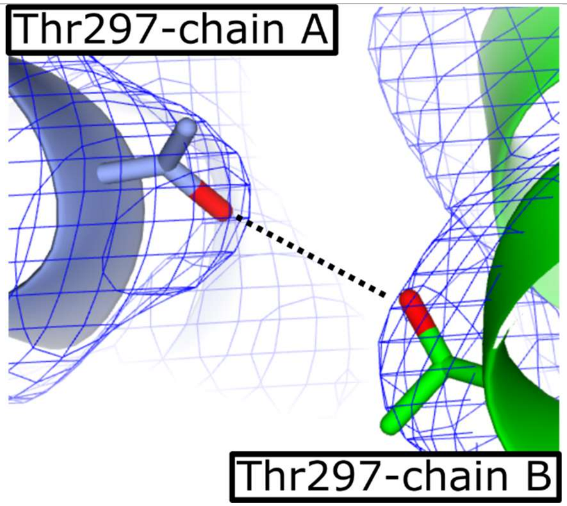	*	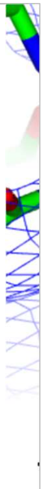
Asp337	loop Aα8-Cα1'	2.71	Lys258	Aα6	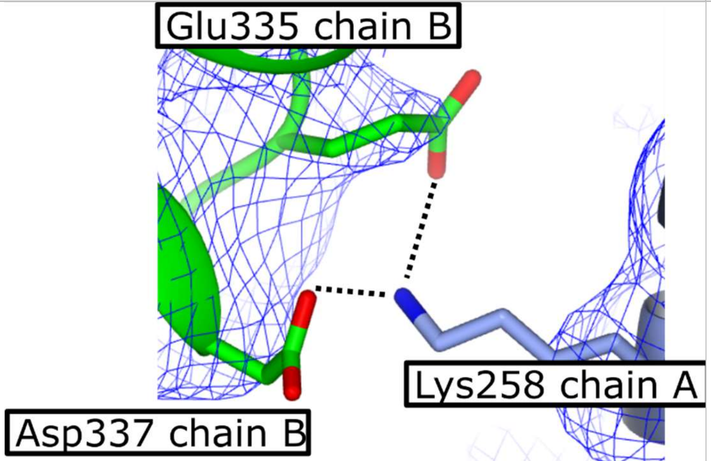	*	

Glu251	Aα6	3.10	Lys327	Aα8	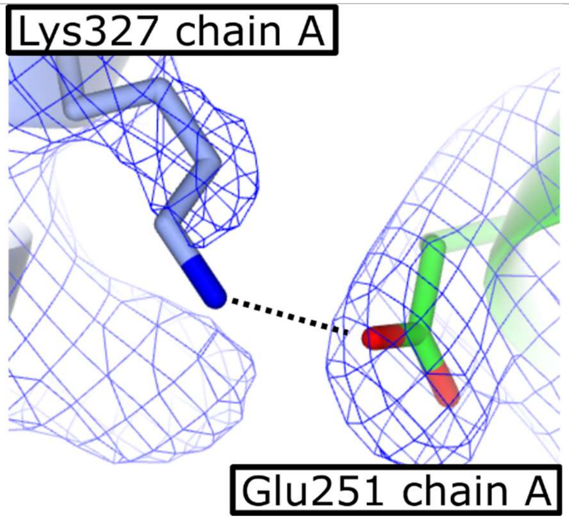	*
Gln341	Cα1’	3.01	Gln341	Cα1’	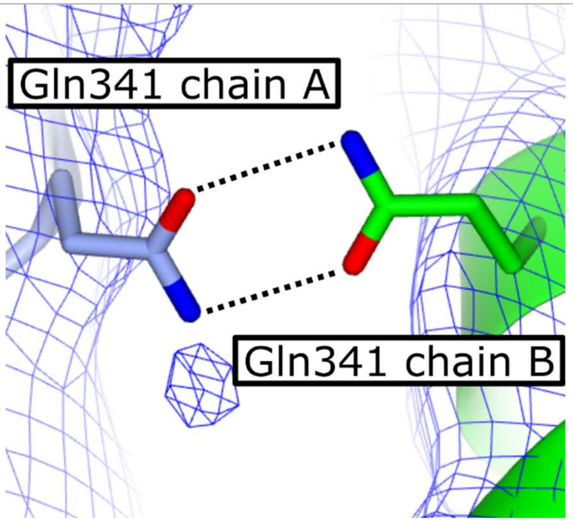	*
	**Interactions at the C-C interface in PykF**					

Chain B	Secondary structure	Distance (Å)	Chain C	Secondary structure		
Ile467	Cβ5	2.91	Asn463	Cβ5	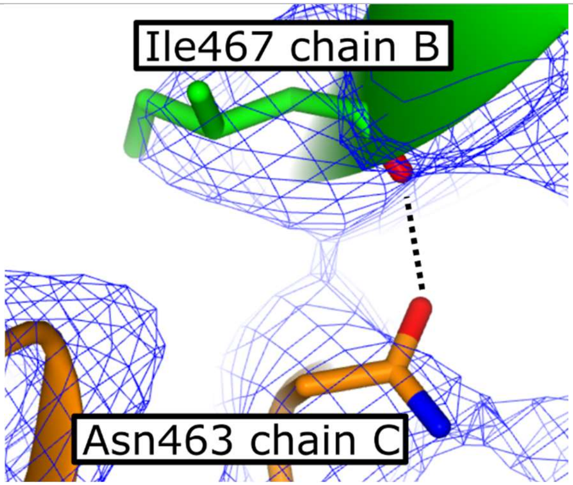	*
Leu465	Cβ5	2.92	Leu465	Cβ5	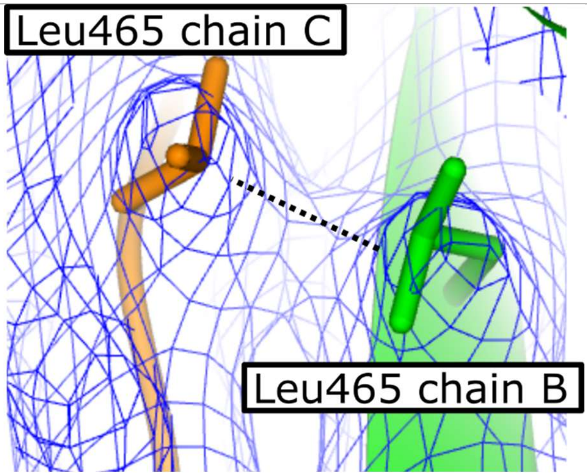	*
						*

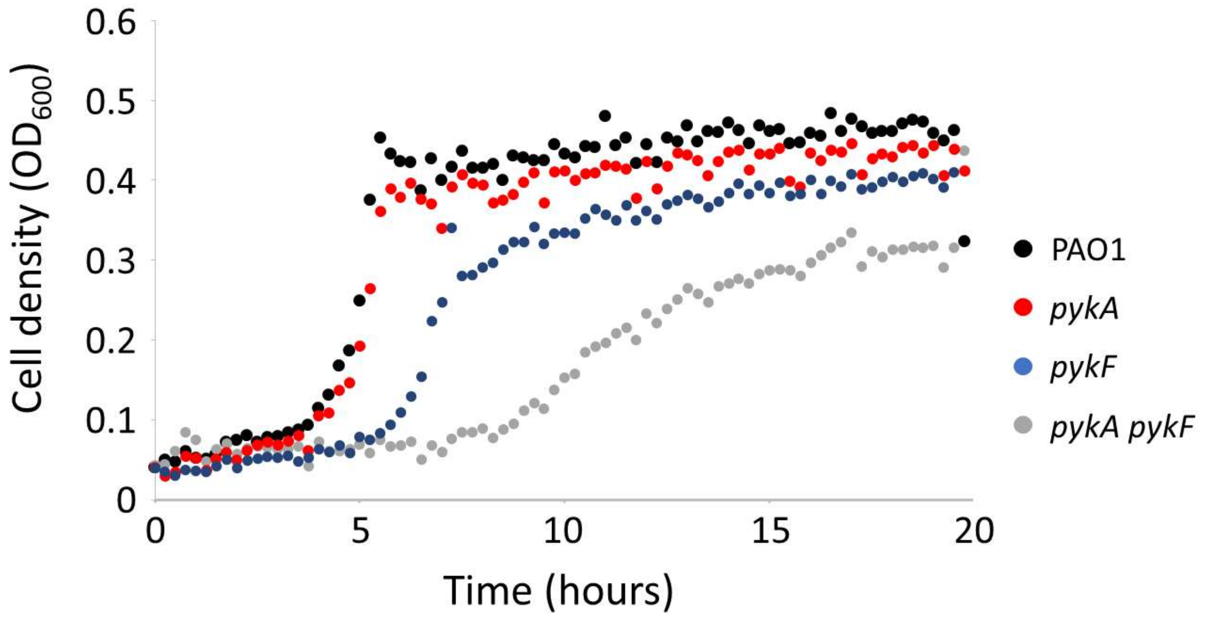

Figure S1. Growth of strains PA01, PW8308 (*pykA*), PW3705 (*pykF*) and PAF0 (*pykA pykF*) in M9 minimal media containing 20.8 mM allantoin at 37°C. Aliquots of culture (100 μL volume) were incubated in a 96-well plate sealed with a gas-permeable membrane in an Omega FLUOstar plate reader. OD_{600} measurements were taken every 15 minutes, with shaking at 200 rpm between readings. Data represent the mean of three biological replicates.

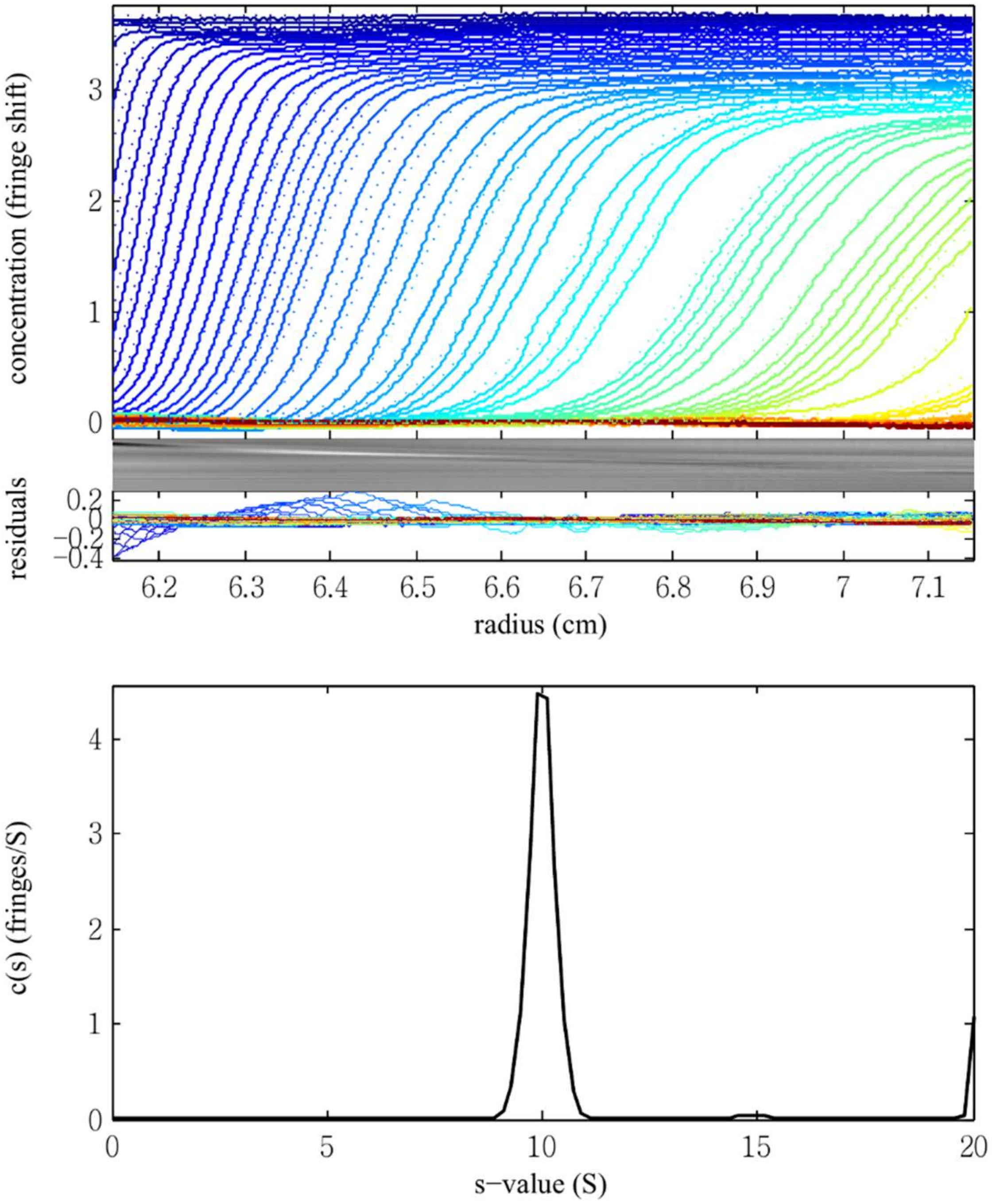

Figure S2. Analytical ultracentrifugation analysis showing that PykF (51.5 kDa/monomer) is a 192 kDa tetramer in solution.

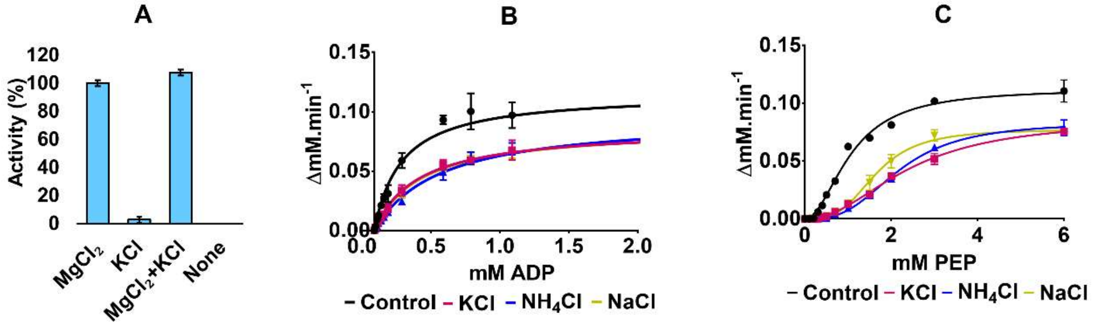

Figure S3. Effects of metal ions on PykF activity. (A) Effects of Mg^{2+} and K^+ on PykF activity. $MgCl_2$ and KCl were added at 10 mM and 100 mM concentration, respectively. PEP and ADP were added at saturating concentrations (5 mM and 2 mM, respectively). (B), (C) Effects of monovalent ions on PykF kinetics. For the ADP titration, PEP was present at a fixed concentration of 5 mM, whereas for titration of PEP, ADP was present at a fixed concentration of 2 mM. Monovalent cations were present at 100 mM concentration. Data points of (A), (B) and (C) represent the mean and standard deviation from three independent experiments.

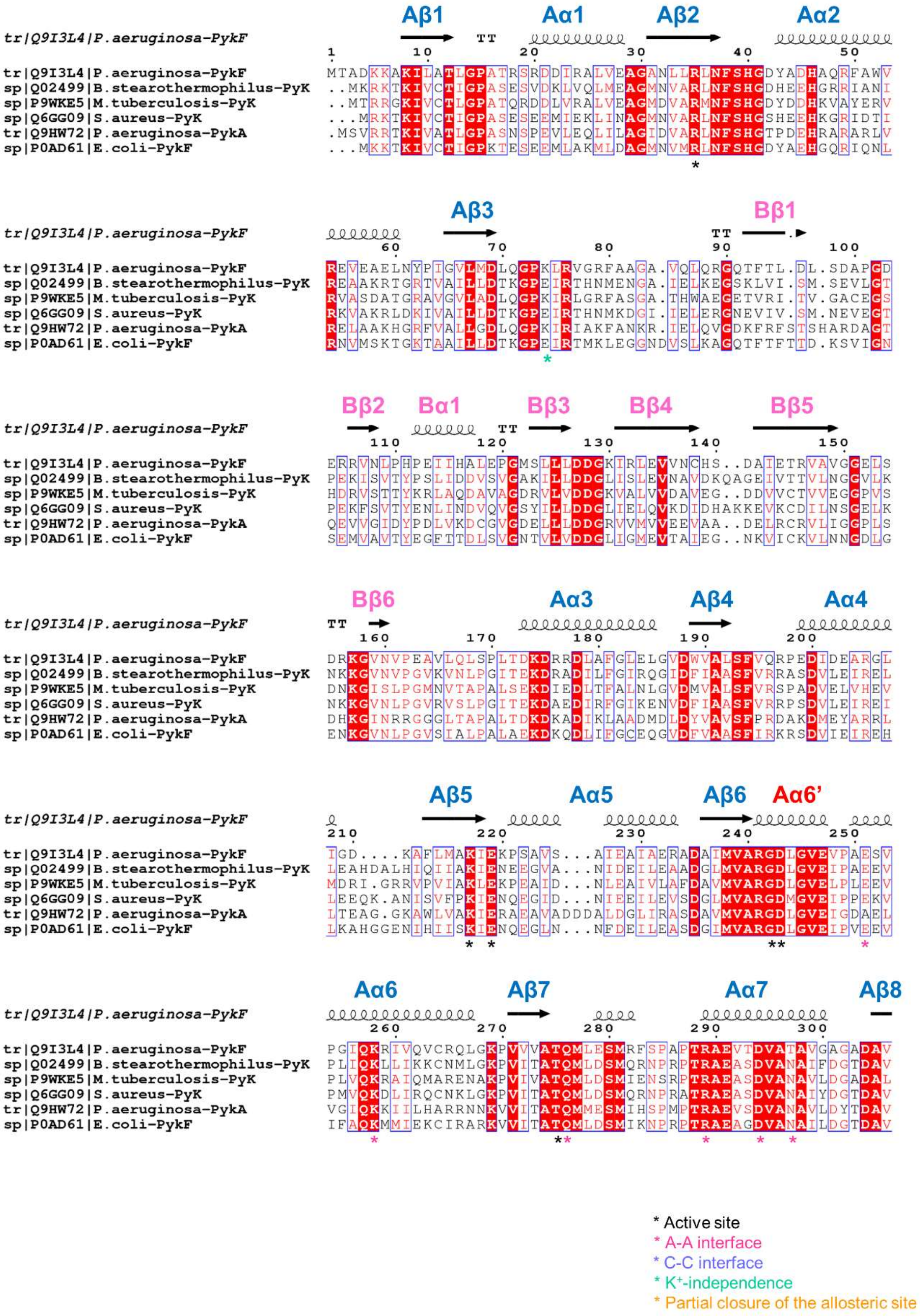

Aβ1 Aα1 Aβ2 Aα2
Aβ3 Bβ1
Bβ2 Bα1 Bβ3 Bβ4 Bβ5
Bβ6 Aα3 Aβ4 Aα4
Aβ5 Aα5 Aβ6 Aα6'
Aα6 Aβ7 Aα7 Aβ8
tr|Q9I3L4|P.aeruginosa-PykF
sp|Q02499|B.stearothermophilus-PyK
sp|P9WKE5|M.tuberculosis-PyK
sp|Q6GG09|S.aureus-PyK
tr|Q9HW72|P.aeruginosa-PykA
sp|P0AD61|E.coli-PykF
* Active site
* A-A interface
* C-C interface
* K+-independence
* Partial closure of the allosteric site

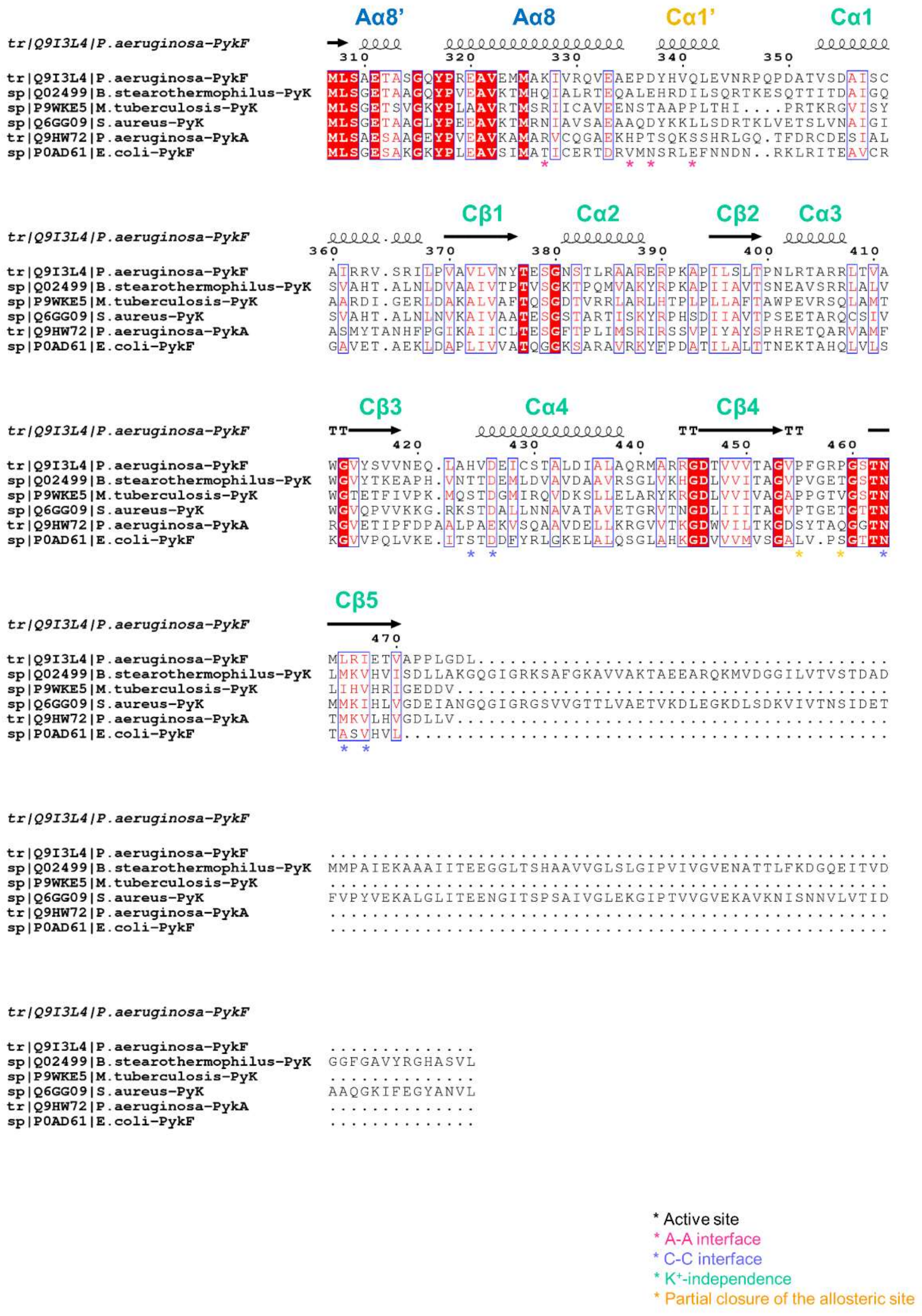

Figure S4. **Amino acid sequence alignment of PK in bacterial species.** The figure shows a sequence lineup of different bacterial PK isoforms for which structural information is available through the Protein Data Bank. Large-scale differences between these structures are summarized in **Table 3**. Amino acid numbering and secondary structures refer to *P. aeruginosa* PykF. Secondary structures of the A, B and the C domains are coloured in blue, pink and green, respectively, with the exception of Cα1’, which is coloured in orange. Absolutely conserved amino acids are highlighted in red whereas highly-conserved blocks of sequence are boxed in blue. Coloured asterisks below the sequence represent residues that are associated with the active site (black), A-A interface (pink), C-C interface (blue), K^+-independence (green) and the partial closure of the allosteric site (orange).

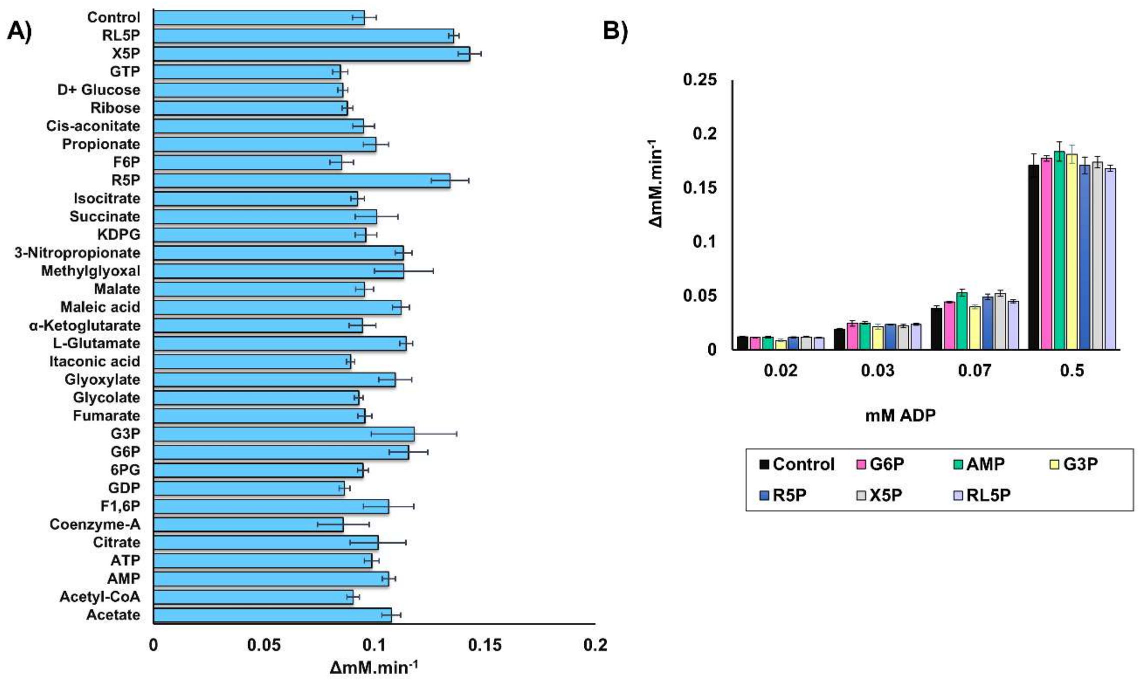

Figure S5. (A) The effect of different metabolic regulators on PykF activity at high [PEP] (3mM) and 2 mM ADP. Putative regulators were added at 1 mM final concentration, except for R5P, X5P and RL5P which were used at 0.15 mM, 0.5 mM and 0.5 mM, respectively. The aim of this experiment was to identify potential allosteric inhibitors. (B) ADP-dependency of potential regulators of PykF. PykF activity was measured in the presence of 1 mM G3P, 1 mM G6P, 1 mM AMP, 0.15 mM R5P, 0.5 mM X5P and 0.5 mM RL5P. PEP was added at 5 mM concentration. Data of figures (A) and (B) represent the mean and standard deviation of three independent experiments.

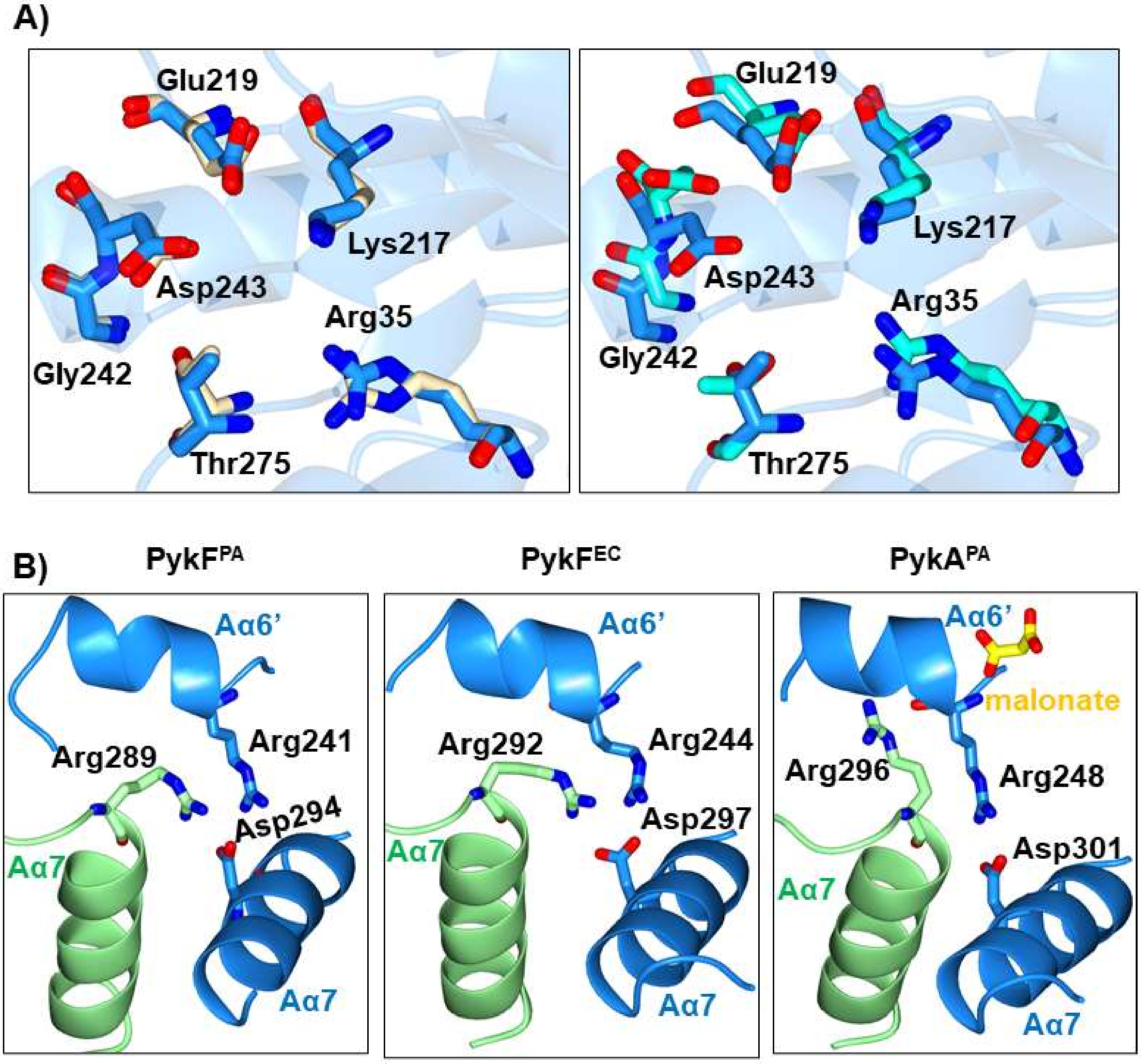

Figure S6. The active site of PykF. (A) Inactive conformation of the active site of PykF. (left panel) Superposition of the side chains present in active site of $PykF_{PA}$ (blue) and $PykF_{EC}$ (1PKY, yellow). (right panel) Superposition of the side chains present in active site of $PykF_{PA}$ (blue) and $PykA_{PA}$ (6QXL, cyan). The numbering of residues refers to *P. aeruginosa* PykF. (B) Close-up views of the active site in $PykF_{PA}$, $PykF_{EC}$ (1PKY) and $PykA_{PA}$ (6QXL) showing that Arg289 of $PykF_{PA}$ and Arg292 (equivalent to Arg289 in $PykF_{EC}$) orient away from the active site, whereas Arg296 (equivalent to Arg289 in $PykA_{PA}$) orients towards the active site.

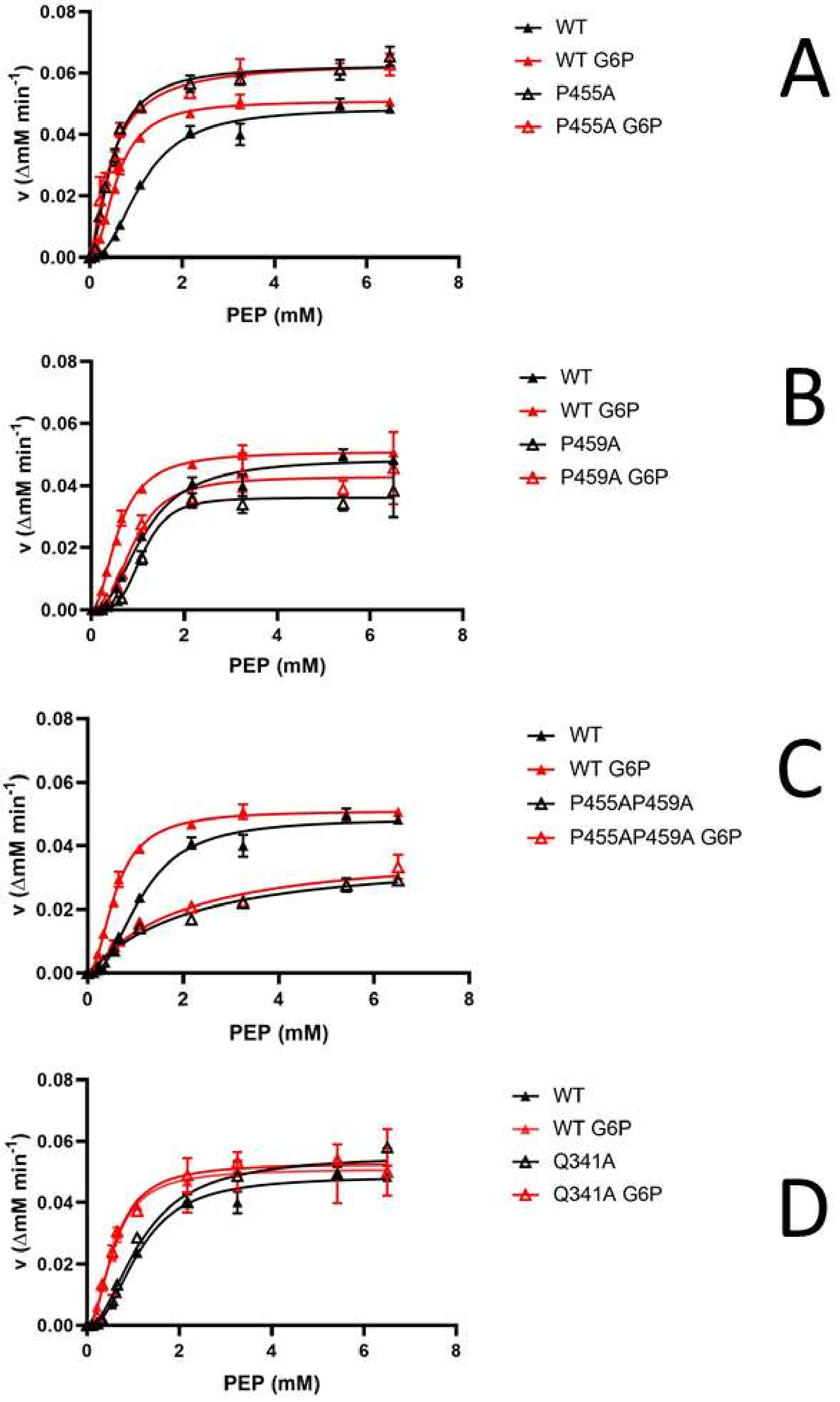

Figure S7. Kinetic analyses of the (A) P455A, (B) P459A, (C) P455A P459A double mutant, and (D) Q341A mutant PykF proteins. In each case, we also show the kinetics of a preparation of the wild-type protein (± 1 mM G6P) for comparison. Kinetics assays were performed at 37°C in a 200 uL reaction mixture containing 50 mM Tris-HCl, 10 mM $MgCl_2$, 20 units of rabbit muscle LDH, 0.2 mM NADH, 0.25 μg/mL of PykF enzyme and 0-6.5 mM PEP. Where indicated, G6P was added at 1 mM final concentration. Data represent the means and standard deviations observed in three independent experiments.

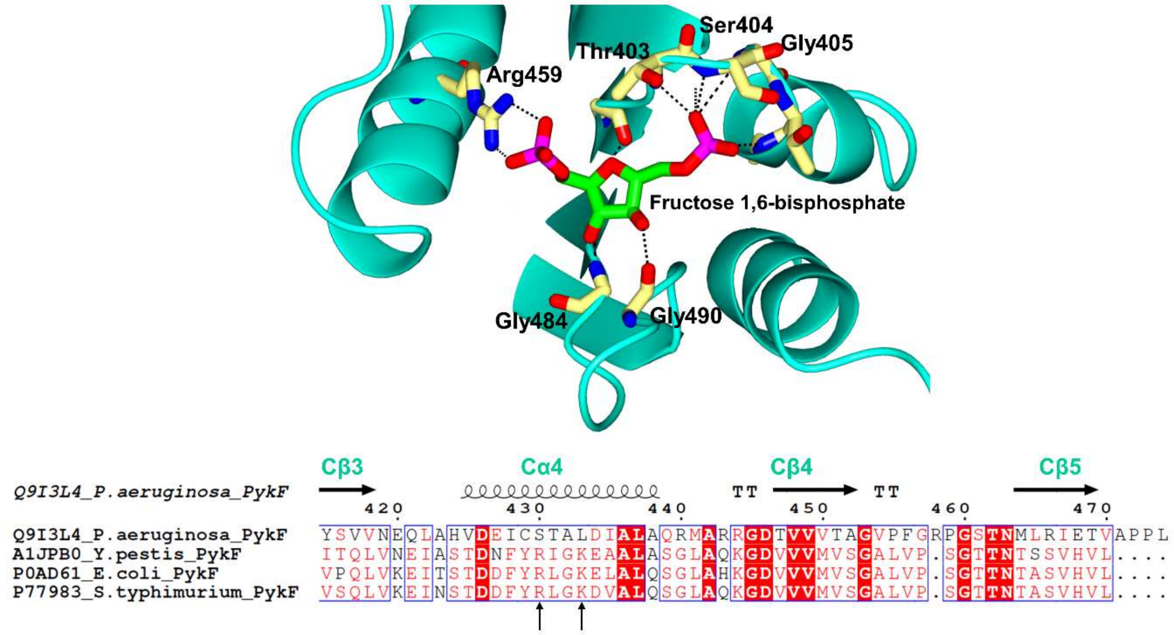

Figure S8. (Top) A close-up view of the allosteric site of yeast PK (PDB 1A3W) showing that an arginine from Cα4 (Arg459) is required to facilitate binding of the 1-phosphate moiety in fructose 1,6-*bis*phosphate to the allosteric site. (Bottom) Amino acid sequence alignment of *P. aeruginosa* PykF and other bacterial PykF isoforms that are regulated by fructose 1,6-*bis*phosphate. The black arrows point to the residues that likely facilitate the binding of fructose 1,6-*bis*phosphate to the enzymes in these species. Note that PykF from *P. aeruginosa* lacks these residues.

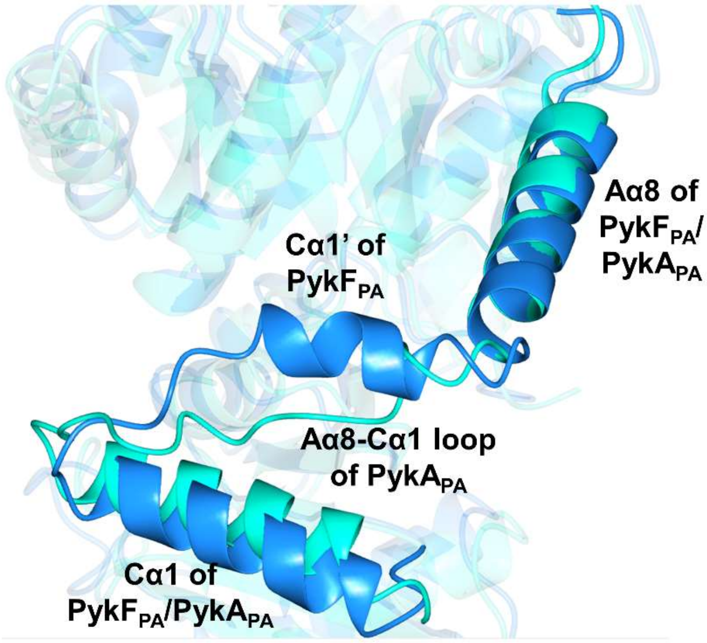

Figure S9. Superposition of the Cα1- Cα1'-Aα8 region of PykF$_{PA}$ with the analogous structures (Cα1- Aα8/ Cα1 loop- Aα8 region of PykA$_{PA}$).
